# Tea and tea drinking: China’s outstanding contributions to the mankind

**DOI:** 10.1186/s13020-022-00571-1

**Published:** 2022-02-22

**Authors:** Si-Yuan Pan, Qu Nie, Hai-Chuan Tai, Xue-Lan Song, Yu-Fan Tong, Long-Jian-Feng Zhang, Xue-Wei Wu, Zhao-Heng Lin, Yong-Yu Zhang, Du-Yun Ye, Yi Zhang, Xiao-Yan Wang, Pei-Li Zhu, Zhu-Sheng Chu, Zhi-Ling Yu, Chun Liang

**Affiliations:** 1 School of Traditional Dai-Thai Medicine, West Yunnan University of Applied Sciences, Jinghong, Yunnan China; 2 School of Chinese Materia Medica, Beijing University of Chinese Medicine, Beijing, China; 3 School of Chinese Medicine, Hong Kong Baptist University, Hong Kong, China; 4 Division of Life Science, Center for Cancer Research, and State Key Lab of Molecular Neuroscience, Hong Kong University of Science and Technology, Hong Kong, China; 5 EnKang Pharmaceuticals (Guangzhou) Ltd, Guangzhou, China

**Keywords:** Tea, Tea beverage, Tea culture, Human health, COVID-19, Harmonious society

## Abstract

**Background:**

Tea trees originated in southwest China 60 million or 70 million years ago. Written records show that Chinese ancestors had begun drinking tea over 3000 years ago. Nowadays, with the aging of populations worldwide and more people suffering from non-communicable diseases or poor health, tea beverages have become an inexpensive and fine complementary and alternative medicine (CAM) therapy. At present, there are 3 billion people who like to drink tea in the world, but few of them actually understand tea, especially on its development process and the spiritual and cultural connotations.

**Methods:**

We searched PubMed, Google Scholar, Web of Science, CNKI, and other relevant platforms with the key word “tea”, and reviewed and analyzed tea-related literatures and pictures in the past 40 years about tea’s history, culture, customs, experimental studies, and markets.

**Results:**

China is the hometown of tea, tea trees, tea drinking, and tea culture. China has the oldest wild and planted tea trees in the world, fossil of a tea leaf from 35,400,000 years ago, and abundant tea-related literatures and art works. Moreover, tea may be the first Chinese herbal medicine (CHM) used by Chinese people in ancient times. Tea drinking has many benefits to our physical health via its antioxidant, anti-inflammatory, immuno-regulatory, anticancer, cardiovascular-protective, anti-diabetic, and anti-obesity activities. At the moment, COVID-19 is wreaking havoc across the globe and causing severe damages to people’s health and lives. Tea has anti-COVID-19 functions via the enhancement of the innate immune response and inhibition of viral growth. Besides, drinking tea can allow people to acquire a peaceful, relaxed, refreshed and cheerful enjoyment, and even longevity. According to the meridian theory of traditional Chinese medicine, different kinds of tea can activate different meridian systems in the human body. At present, black tea (fermented tea) and green tea (non-fermented tea) are the most popular in the world. Black tea accounts for over 90% of all teas sold in western countries. The world’s top-grade black teas include *Qi Men* black in China, Darjeeling and Assam black tea in India, and Uva black tea in Sri Lanka. However, all top ten famous green teas in the world are produced in China, and *Xi Hu Long Jing* tea is the most famous among all green teas. More than 700 different kinds of components and 27 mineral elements can be found in tea. Tea polyphenols and theaflavin/thearubigins are considered to be the major bioactive components of black tea and green tea, respectively. Overly strong or overheated tea liquid should be avoided when drinking tea.

**Conclusions:**

Today, CAM provides an array of treatment modalities for the health promotion in both developed and developing countries all over the world. Tea drinking, a simple herb-based CAM therapy, has become a popular man-made non-alcoholic beverage widely consumed worldwide, and it can improve the growth of economy as well. Tea can improve our physical and mental health and promote the harmonious development of society through its chemical and cultural elements.

## Background

Only tea succeeded in conquering all over the world—Alan Macfarlane

Tea (English), 荼/茶(Chinese), чaй (Russian), thé (French), tee (German), お茶/茶の木 (Japanese), شاي (Arabic), چائے (Urdu), chai (Hindi), or çay (Turkish), an important contributor to population health, economics, and cultural values from China, refers to the cured leaves from the perennial plant *Camellia sinensis* (L.) O. Ktze (scientific name *Camellia sinensis*) through various processing technologies [1, 2]. Generally, tea as a beverage is made as a liquid prepared by mixing hot water with dried tea leaves or extracts obtained from tea leaves. Other than plain water, tea is the most popular and the cheapest man-made non-alcoholic beverage widely consumed all over the world. At present, there are more than 3 billion people who like to drink tea as a beverage in 160 countries and regions. Therefore, tea improves the economic growth around the world. The global tea market was valued at nearly 200 billion U.S. dollars in 2020, and is expected to rise to over 318 billion U.S. dollars by 2025.

Tea-consumers believe that tea has the functions of prolonging life, improving health, and thwarting diseases such as peroxide-associated diseases, cardiovascular diseases, immuno-related diseases, hepatic diseases, diabetes mellitus, inflammation, cancer, obesity, etc. [3–5]. Moreover, they can enjoy the cheerful feelings of tea-drinking processes (tea art/culture). For this reason, more than two billion cups of tea are consumed every day around the world. Today, although there are a lot of modern beverages in the market, tea is still the national beverage, and it spreads every corner of China from important parties to public daily lives as the part of Chinese culture and lifestyle. In both the old and current China, tea drinking can be regarded as a show of personal morality, education, refinement, and status, because of the cultural elements present in tea. Therefore, tea helps to make the Chinese literature, art, philosophy and religions (Taoism, Buddhism and Confucianism) connect inevitably to each other.

For the thousands of years since Chinese tea was discovered by *Shen Nong*, the people of the world have been drinking tea for stimulation, relaxation and aspiration of achieving good health. Tea has become the world's favorite drink. Moreover, the discovery of tea affected the progress of the world history and Chinese history, fueled the industrial revolution and sealed the fate of the modern world. For instance, the first Opium War (1840–1842) and the second Opium War (1856–1850) happened in China and the War of Independence (1875–1873) happened in the USA were all directly or indirectly related to tea [6, 7]. Before 1650, the European tea trade was totally dominated by the Dutch. Afterwards, the British gradually got rid of the monopoly of Dutch on tea trade via twice Anglo-Dutch wars happened in 1652–1667 [8, 9]. After that, Britain became the empire on which the sun did not set for a century. Additionally, tea also alter our life-styles, religions, aesthetics, and etiquette, and in the meantime, it makes our daily lives much more colorful.

Today, you may reject drinking coffee, Coca-Cola and other modern beverages with various different reasons, including the reasons for religious belief, but nobody can refuse to drinking tea. Besides the history, culture and being safe to humans, tea-drinking shows various beneficial effects on human health, including antioxidant, anti-inflammatory, immuno-regulatory, anticancer, cardiovascular-protective, anti-diabetic, anti-obesity, and hepato-protective effects, and even longevity [5, 10–12]. Recently, due to the coronavirus disease 2019 (COVID-19), predictably, tea has gained more fame, because teas have anti-COVID-19 functions via anti-oxidant, anti-stress, anti-inflammatory, anti-virus, and anti-immune-mediated injury activities, as well as polyphenols derived from tea that are potential candidates in prophylaxis and treatment of COVID-19 [13–19]. Although there are 3 billion people who like to drink tea in the world, few of them actually understand tea. In the current paper, we describe the past and present states of tea and emphasize the beneficial effects of tea on human physical and psychological health and the sustainable and healthy development of human society.

Tea has occupied a part of the human heart and is a daily necessity nowadays.

## NPQs in China

Archaeology has proved that there are ten thousand years of Chinese civilization/culture rather than five thousand years as stated in the book *The Records of the Grand Historian* (*Shi Ji* in Chinese) written by *Si Ma Qian* (145–87 BCE), a Chinese historian of the *Han* dynasty (202 BCE-220) [20–23]. Compared with other ancient civilizations in the world that have withered and/or perished, the culture of China is continuing from it ancient times to the present, especially in its language and literature. For example, British people who live in England, Scotland, Wales, Northern Ireland at the moment do not understand the English in Shakespeare's time. However, Chinese characters, except for the Oracle Bone Inscriptions (*Jia Gu Wen* in Chinese), did not change significantly since they were born [24], and ordinary Chinese people nowadays still write and comprehend our ancestors’ languages. For example, *the Yellow Emperor's Classic of Internal Medicine* (*Huang Di Nei Jīng* in Chinese), an ancient treatise on health and disease said to be written around 2000 years ago, is still in print. Although different historical periods have different dominators (emperors) and names that represented various interest groups, the history of China never breaks off. During the 20th country, China was aggressed by imperialism from time to time, but China only became a semi-feudal and semi-colonial country rather than a full-colonial one. Our nation still stands erect the east of the world. Today, China is the only country whose culture is continuing among the Four Great Ancient Civilizations. Unfortunately, Chinese traditional civilization suffered from the destructive effects of the Great Cultural Revolution (1966–1977). Fortunately, for the past 40 years, not only is China advancing rapidly in science and industry, but China’s traditional civilization is also gradually recovered.

There are about two hundred innovations/creations that have played crucial roles in the progress and evolution of society civilization of the mankind. About half of these innovations/creations were made by ancient Chinese. These included the Four Great Inventions: paper making created by *Cai Lun* (48 or 61–121), compass invented by *Wang Chong* (27–97 or 107), gunpowder invented by Taoists in the ninth century, and typography established by *Bi Sheng* (?–1051) [25]. When Marco Polo (1254–1324), a Venetian merchant traveler, reached in China in 1275, he was astonished by what he saw [26]. At the same time, Europeans were also amazed and impressed by a glorious Eastern world (China) as described in the book *The Travels of Marco Polo* written in 1295 [27]. Therefore, we may say that China was the pace-setter not only in culture but also in science and technology during the pre-industrial revolution. The book *Science and Civilization in China* edited by Joseph Needham (1900–1995) recorded the scientific and technological achievements of ancient China.

**Fig. 1:**
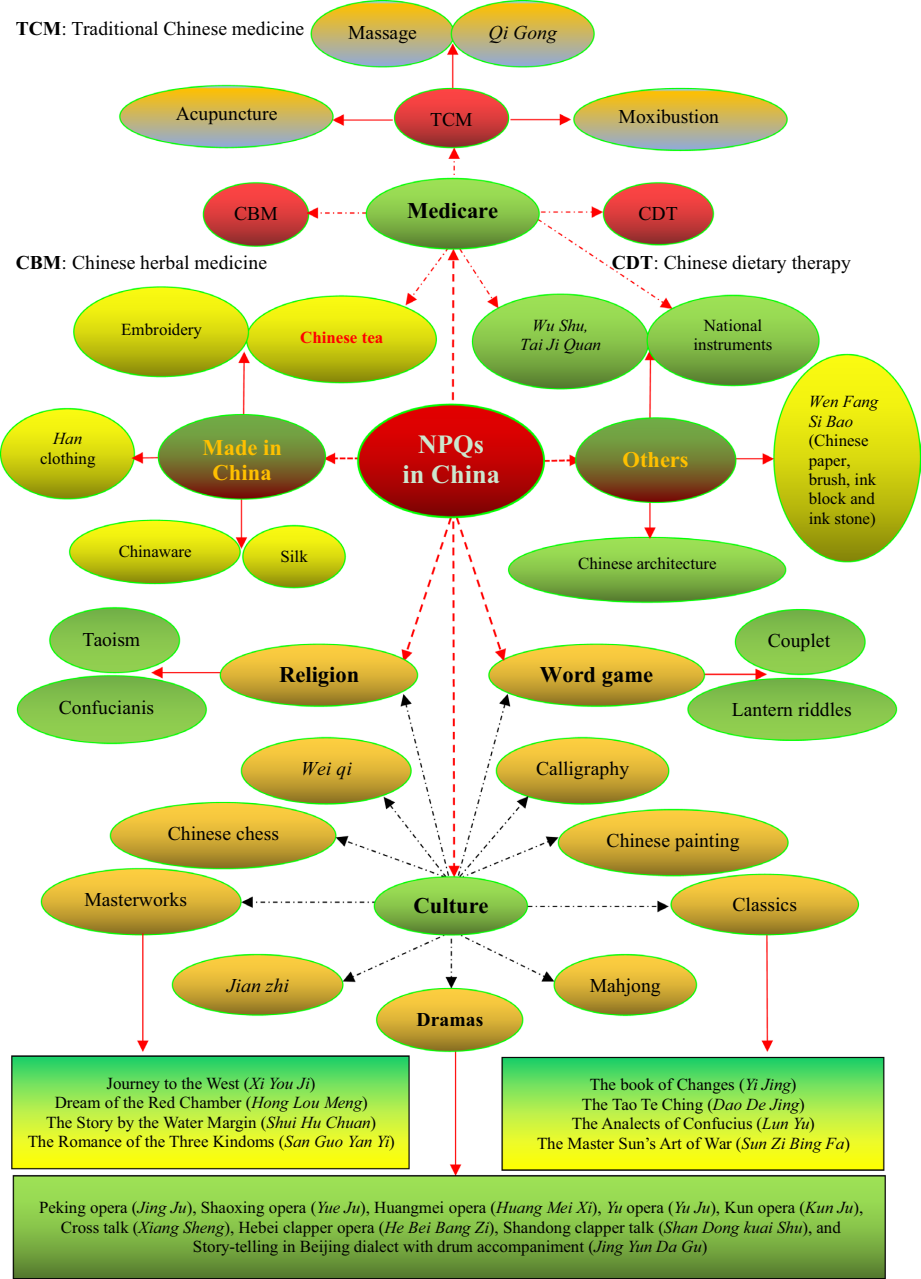
The national peculiar quintessences/treasures (NPQs, 国粹) in China

Because of the abundant humanistic feelings in the ancient culture of China, the ancient innovations and findings that are named the national peculiar quintessence/treasures (NPQ, *Guo Cui* in Chinese, 国粹) still affect our daily lives, especially on Chinese herbal medicine (CHM) and Chinese tea originated in China (Fig. 1) [28, 29]. These NPQs in China can still directly and indirectly help to improve public health by acting through mood (spirit), body (physiology), and even the integration of both. Therefore, they are warmly welcomed by the people in the modern world. For example, more than 8,000 varieties of CHM or CHM-related products are exported from China to more than 130 countries/regions, with more than 50 kinds of CHM exported to the United States annually [30]. Traditional Chinese therapies are also widely used as a complementary and/or alternative medicine worldwide [31]. They are still benefiting the mankind. For example, it is inseparable from the Chinese traditional medicine, culture, values and dedication that the Chinese people have made outstanding achievements in the fight against the COVID-19 pandemic [32–34]. This epidemic has demonstrated to the world the advantages of the Chinese culture and system, which will affect the future trend of the world pattern.

Will China save the world? This may be true.

## China, the birthplace of tea

It has been demonstrated that the tea plants distributed around the world are native to southwest China based on a large amount of historical data/documents, as well as modern research findings. According to cluster analysis, various types of intermediate hybrids and spontaneous polyploids are found because tea trees can easily hybridize, and there is likely just a single place/area of origin for *Camellia sinensis* on earth. This place is believed to be the adjoining area of the *Yun Nan* and *Si Chuan* provinces of China, India, and northern Burma [35].

### Tea-related resources in China

In both the ancient and modern times, the development of human civilization, including science, technology and culture, is closely related to the conditions of natural geography environment, namely the unity of man and nature, said by the ancient Chinese.

#### Geography

Tea trees are tropical evergreen shrubs, small trees/arbors, and trees/arbors, plants up to 1–6 m even 15–30 m tall, that are grown mainly between 30 and 16 degrees north–south latitude of the equator. Generally, the finest tea is grown at altitudes of 900 to 2100 m. *Heng Duan* Mountains and *Yun Nan-Gui Zhou* Plateau located in southwest China are the cradle of tea plants, where there are still many living wild tea trees that are hundreds and even thousands of years old. The growth of tea shrubs requires acid soil and a warm/moist setting with at least 50 inches of annual rainfall. Southern China, including 15 administrative provinces and autonomous regions, locates in the subtropical zone, with warm and moist climate ideal for tea plants. The rest of China is not suitable for growing tea trees, due to the low/high temperature in winter/summer, persistent drought, or a combination of the above. However, 58 kinds of cold-resistant tea trees are cultivated in China. Therefore, it is no doubt that China’s tea species and commercial teas are the most abundant in the world.

#### Tea trees

**Fig. 2:**
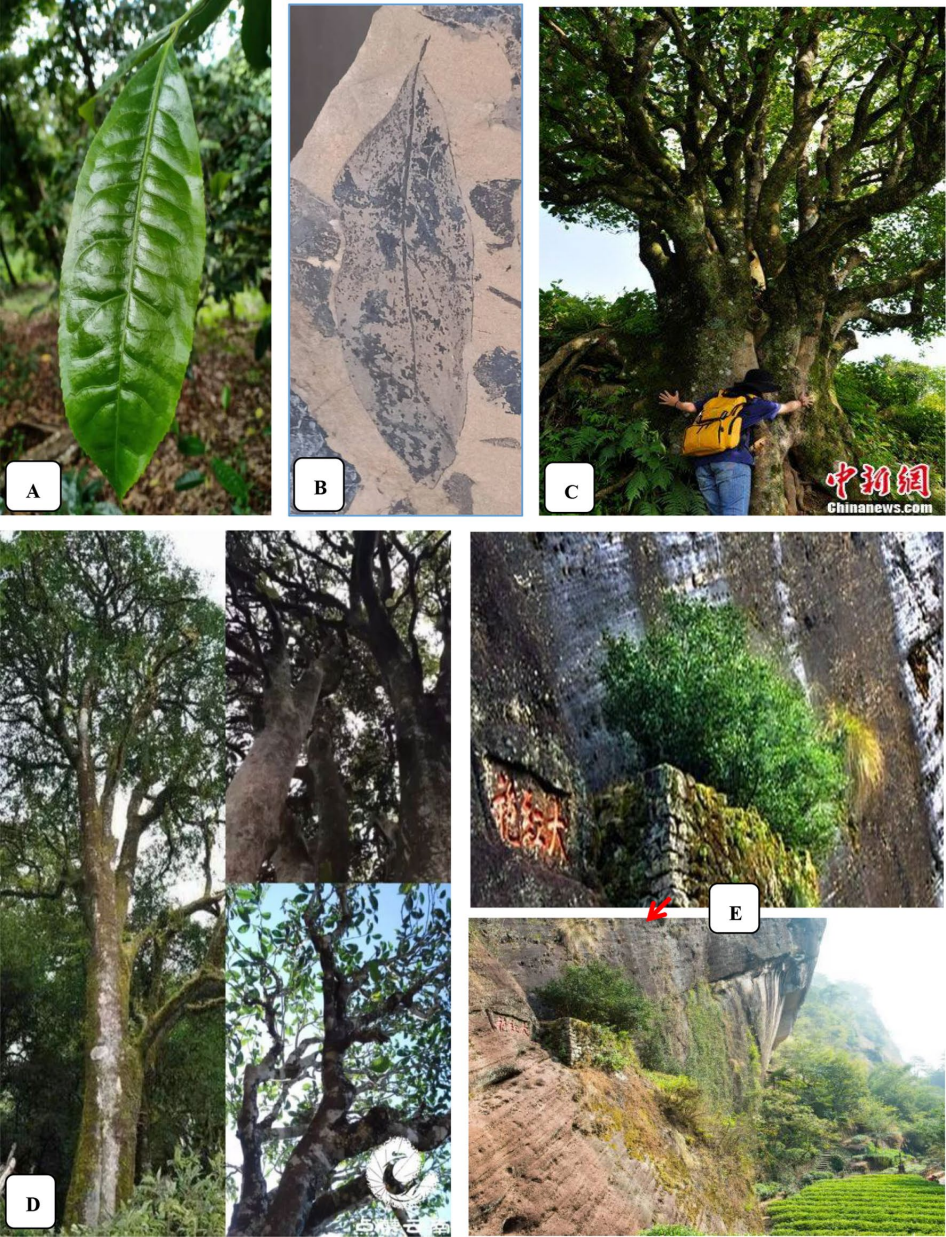
Current tea leaf, fossil of tea leaf, and the oldest tea tree in China. **A** Current tea leaf; **B** Fossil of tea leaf in 35,400,000 years ago; **C** 3200-year-old planted. Data (**C**, **D**, **E**) from http://www.chinanews.com/tp/2012/08-10/4098604_2.shtml#nextpage and http://www.360doc.com/content/15/0506/09/2788370_468401856.shtml

There is rich germplasm resource of natural and cultivated tea trees in the west coast of the Pacific Ocean in China. More than 380 species of theaceae plants belonging to 23 families are found in the world, of which more than 260 species belong to 15 families in China. Most of the Chinese tea species are distributed in *Yun Nan*, *Gui Zhou* and *Si Chuan* areas [36–38]. In addition, more than 300 kinds of tea trees have been cultivated in China. The oldest (2700-year-old) and the biggest wild tea trees and the oldest planted tea trees (3200 years old) in the world are found in the town of *Si Mao* in *Yun Nan* province of China. In 1961, a 1700 year old, 32 m tall and more than one meter wide wild tea tree, called “the king of tea trees”, was found in the rain forest of *Yun Nan* province of China. In 2004, a wild tea tree over 20 m tall with a diameter of 3.5 m was found in *Shuang Jiang* county of *Yun Nan* province, which may be the oldest or the second oldest wild tea tree on earth [39, 40]. In 1978, fossil of Chinese tea lived 35,400,000 years ago was discovered in *Si Mao* in *Yun Nan* province of China. In addition, 196 places with wild tea trees distributed in the southern and southwestern areas of China have been discovered, and some of the trees reach 25 m tall. These tea trees are of typical primitive forms, such as *Polyspora atillaris* (Roxb) Sweet, *Schima wallichii* Choisy, *Eurya acuminatissima* Merr. et Chun, *Ternstroemia gymnanthera* (Wight et Arn.) Sprague, *Tutcheria spectabilis* Dunn and *Camellia* plants *C. muricatala* Chang, sp. Nov., *C. albovillosa* Hu, sp. Nov., *C. olifera* Abel, *C. japounica* Linn., *C. pachyandra* Hu, etc. (Fig. 2).

Although Bruche R. found wild tea trees in Sadiya region in India in 1824, research has demonstrated that tea trees in India, Taiwan and Burma originated from *Yun Nan* and *Si Chuan* areas in China. However, it has also been suggested that India may be a possible origin place of the Indian Assam tea, a type of big-leaved tea [41–43]. However, recent research showed that there were two origins of Chinese Assam type tea, namely, the southern and western areas of *Yun Nan* in China, and the Chinese Assam type tea diverged 22,000 year ago and split into the Chinese Assam type tea and Indian Assam type tea [44]. Moreover, statistical cluster analysis and the findings on the chromosome numbers, the nature of easy hybridization, and the existence of various types of intermediate hybrids and spontaneous polyploids indicate that a single place of origin likely exists for *Camellia sinensis* in an area between the northern part of Burma and *Yun Nan* and *Si Chuan* of China. Therefore, it is likely that China type tea, China and Indian Assam type tea, and most of the widely distributed and planted tea trees in the world originated in China. Furthermore, tea trees were also originally discovered, applied and planted (beginning more than 2000 years ago, even 6000 years ago) by Chinese people. Although the wild ancestor of tea plants has not been discovered in China up to now [45], there are plenty of the world’s oldest wild and cultivated tea trees in China. Modern genetic study may provide clues to how tea plants were first domesticated [46].

#### Tea industry and cultivation

**Fig. 3:**
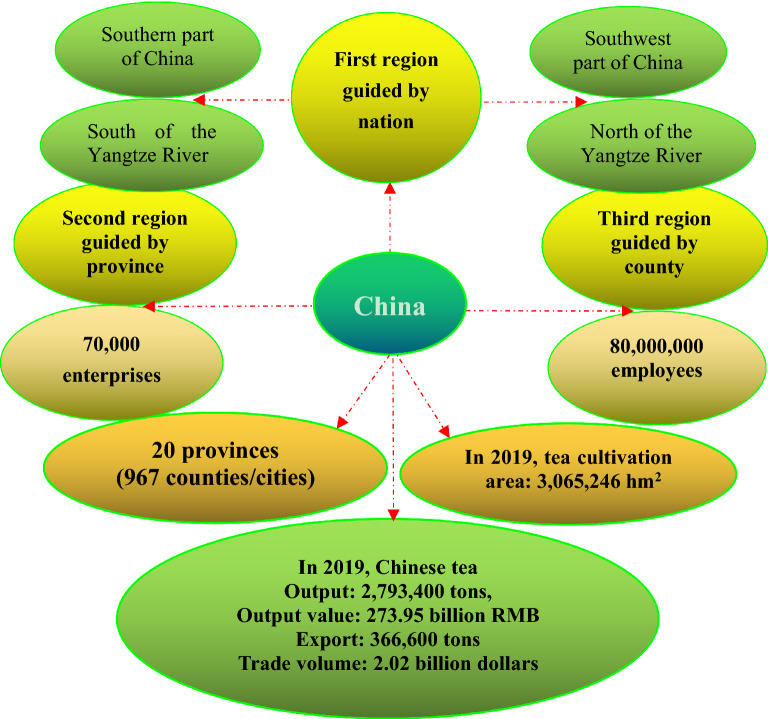
Current status of tea industry and tea cultivation in China

The global tea production amounted to approximately 5,800,000 tons in 2018, and it is estimated that the global tea market will be worth around 81.6 billion U.S. dollars in 2026. China produced more than 2,790,000 tons of tea in 2019, up from about 1,020,000 tons in 2006. In 2018, the global export quantity came to about 1,760,000 tons, and the largest tea-exporting countries include Indonesia, India, China, Sri Lanka and Kenya. In 2020, China exported approximately 2.04 billion U.S. dollar worth of tea, making it the leading exporter of tea worldwide [47]. Other major tea exporters included Sri Lanka and India that year. At present, there are more than 70,000 tea-related enterprises and more than 80,000,000 employees. There are three tea-producing regions including 20 provinces in China. In 2019, the tea-planting areas in China produced 61% of the global total, with India at 15% and Sri Lanka at 5% (Fig. 3).

#### Tea literatures

**Fig. 4:**
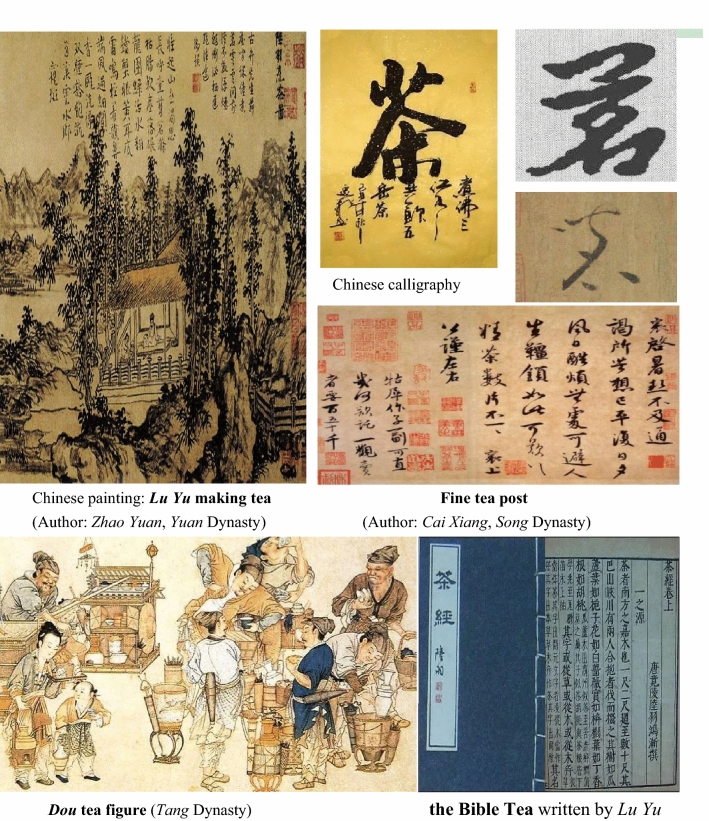
Tea painting and calligraphy in China. Data from https://www.sohu.com/a/296056238_817830, https://baike.so.com/gallery/list?ghid=first&pic_idx=1&eid=5396387&sid=5633577, and https://baike.so.com/gallery/list?ghid=first&pic_idx=1&eid=4525889&sid=4735964

China has the oldest and the richest tea-related literatures in the world (Fig. 4). *The Bible of Tea* (*Cha Jing* in Chinese, 茶经*)* written by the “Tea King” *Lu Yu*, is considered as the world’s first monograph about tea. In this book, author explicated the ways to cultivate and drink tea and different classifications of tea in details, as well as changing the Chinese word “荼” to “茶”. In addition, the Chinese words for tea include both “茗” (tender shoots of tea trees) and “茶” (leaves of tea trees). From the *Tang* dynasty (618 CE–907 CE) to the *Qing* dynasty (1644 CE–1911 CE), China produced 19 famous books, 15 famous paintings, 8 famous proses, and 10 famous calligraphies on tea. In addition, more than 2000 poems and songs related to tea were published during this period [48]. In 1685, the book named *The Traites Nouveaux et Curieux du Cafe, du The et du Chocolat*, the first book about tea in Europe, was published in France [49]. Figure 4 shows some of Chinese paintings and calligraphy on tea.

### Tea-consuming history in China

**Fig. 5:**
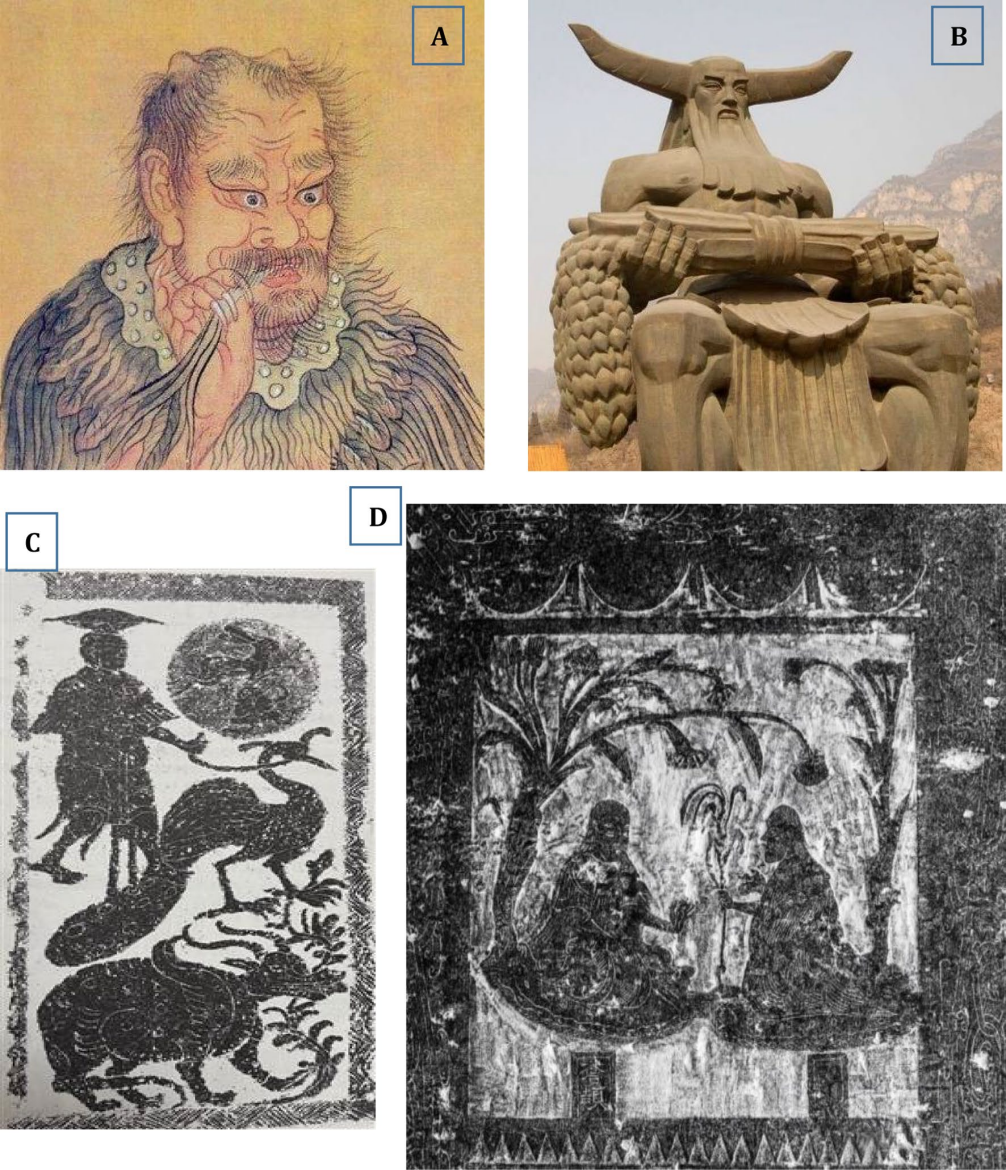
*Shen Nong* (神农)*,* the inventor of agriculture and medicine in China. Data from https://www.360kuai.com/pc/9a0ea12eadc6c1414?cota=3&kuai_so=1 (**A**), https://www.photophoto.cn/tupian/shennongdiaosu.html (**B**), http://www.chinaknowledge.de/History/Myth/personsshennong.html (**C**), and https://w.dzwww.com/p/4848002.html (**D**)

In China, there is a very long history of tea-drinking, with records of its use dating back to the third century, as early as the documented history, i.e., the times of the Three Emperors and Five Sovereigns (三皇五帝 period, 2737–2697 BCE) [50]. In the *Shang* dynasty (1600–1046 BCE), at the latest, ancient Chinese began to drink tea, which was originally used as a medicinal beverage, according to the history record. It is well known that *Shen Nong* (*Shen Nong Shi)*, a clan originated from the agricultural tribes in the ancient *Wu Ling* area about 9000 years ago, was the oldest forefather and the inventor of agriculture and medicine in China (Fig. 5). It was he who first discovered tea in 2737 BCE. “*Shen Nong Shi* personally tasted hundreds of species of herbs and he was hit by 72 poisons in a single day. But he used a kind of tree leaves to ease his poisoning and it turned out to be tea tree” was recorded in a book named *Shen Nong’s Herbal Classic* (*Shen Nong Ben Cao Jing* in Chinese) written in the *Qin/Han* dynasty period (221 BCE–220). This means that the beneficial effects of tea on human being was already realized by Chinese ancestors before *Shen Nong,* and tea might be the first herbal/natural medicine used by the mankind. In fact, the literary tea (荼, *Cha* in Chinese) first appeared in the text of *The Book of Songs* (*Shi Jing* in Chinese) written in the preliminary stage of the *Western Zhou* dynasty (1046–771 BCE). *Shen Nong* was a legendary person, whose achievements and stories guided the evolution and development of Chinese civilization, including the habit of drinking tea.

**Fig. 6:**
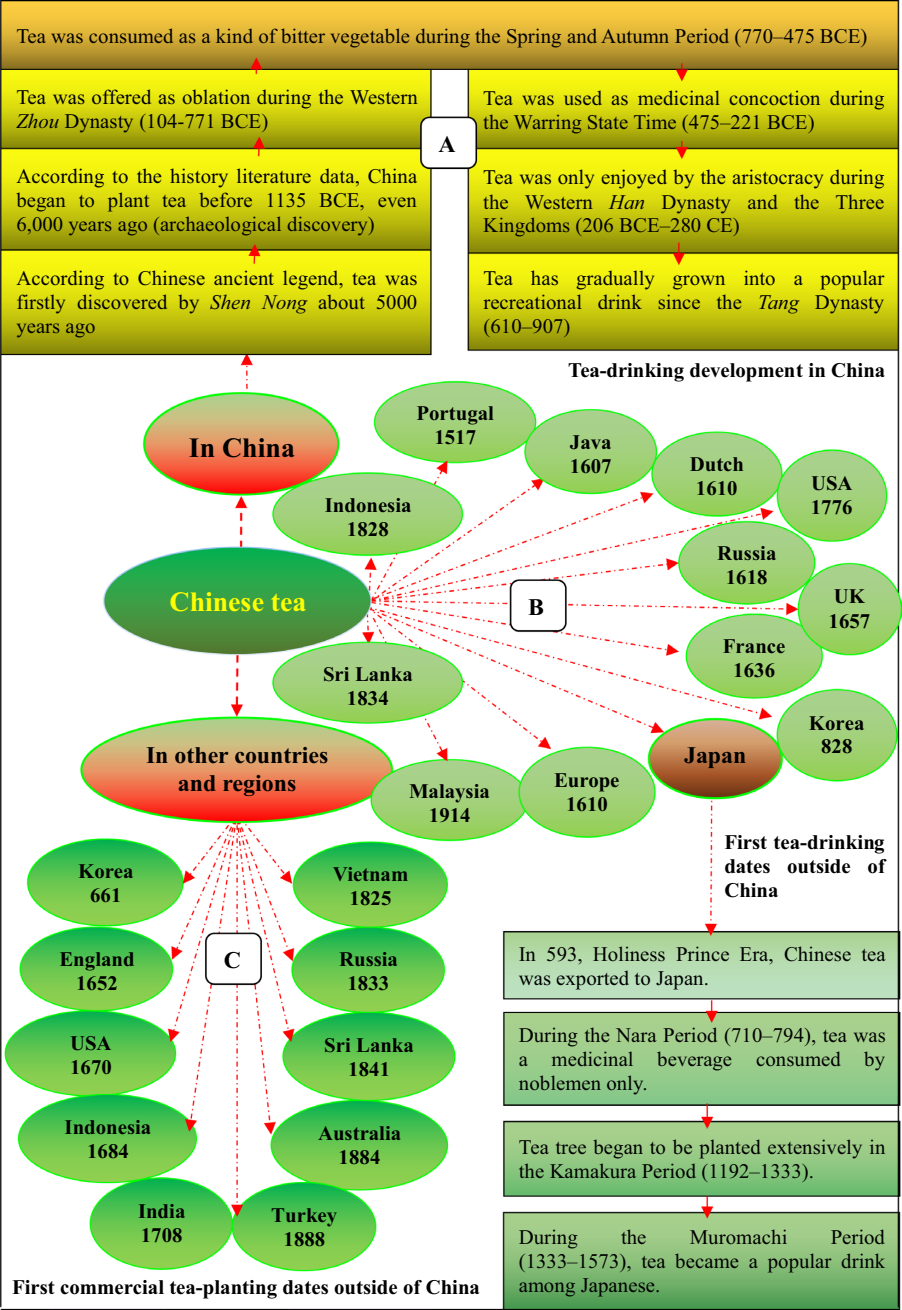
The dissemination of Chinese tea in the world

According to the data recorded in the book *The Classic of Tea*, it is estimated that the discovery and use of tea by ancient Chinese people has a history of nearly 5000 years. During the period of the *Western Zhou* dynasty (1046–771 BCE), tea was employed as oblation. In the *Spring and Autumn* Period (770–476 BCE), tea was a kind of vegetable, “bitter vegetable” (苦菜). Tea drinking for medicinal purposes may have begun in the *Yun Nan* region during the *Shang* dynasty, and was used as medicine to treat some human illness in the *Warring State* Time (475–221 BCE) in China. Before this, however, concentrated liquid from tea leaves without the addition of other leaves or herbs was used as a bitter yet stimulating drink, rather than as a medicinal concoction in *Si Chuan* of China. From the *Western Han* dynasty to the *Three Kingdoms* (206 BCE-280), tea was enjoyed by the aristocracy as a rare and valuable substance. Tea did not become a popular drink enjoyed by the public for recreational use until the *Tang* dynasty (610–907) (Fig. 6A) [51, 52]. Today, drinking tea, like eating food, has become a must in daily lives in both rural and urban China and India. In 2019, 2,069,000 tons of tea leaves was consumed by 490 million Chinese people, which accounted for 36.85% of the total world tea consumption. Although India is one of the largest tea-producing countries in the world, over 70 percent of its tea (approximately 800,000 tons per year) is consumed within India itself.

### Dissemination of Chinese tea

At present, there are more than 60 countries that plant tea trees in tropical and subtropical regions and 3 billion tea drinkers in the world. China and India are ranked first and second tea-producing countries in the world and account for 36.28% and 22.61% of the global tea production, respectively.

#### Tea-drinking dissemination

In the fifth century, during the *Southern and Northern* Dynasties period (420–589), the oversea dissemination of Chinese tea began in 483–493. At that time, tea output was the main goods in the border trade between China and Turkey. Afterwards, tea originated in China, including its planting, processing and drinking arts, along with silk and porcelain, has spread across the world, including other East Asian countries/regions via the Sea-Land Silk Road (beginning during the *Han* dynasty), the Seven Voyages to Western Ocean by *Zheng He* (1371–1433) from 1405 to 1433, and the businessmen in the border trade.

During the *Tang* dynasty, tea-drinking spread to other Asian countries such as Korea, Japan, and Vietnam. In 593, Chinese tea was first exported to Japan along with Chinese culture, art and Buddhism. In 1295, Marco Polo returned to Italia and brought back tea, silk, china, and jewels from China. In 1559, Europeans initially obtained the knowledge about tea via the book *Tea in China and Maritime Travel* written by Lamu Horta. In 1607, tea was shipped from Macau to Java by the Dutch East India Company. In 1609, Chinese tea reached Europe from Hirado of Japan. Tea was first imported into Dutch by the Dutch East India Company in 1610. In 1650, the Dutch East India Company imported Chinese tea to Europe. In 1657, tea began to be sold to the public in England; afterwards the British fell in love with tea, especially after the King Charles II married Portuguese Catherine of Braganza in 1662. It is reported that she brought with her a small chest of tea as part of her dowry and *Qi Men Mao Feng* was one of her teas of choice (Fig. 6B) [53]. In the *Ming* dynasty (1368–1644), tea was sold to South African countries. In 1868, China exported 871,047 tons of tea, worth 37,172,012 taels of silver, accounting for 53.8% of the total export value of all commodities in that year. The East India Company imported 30,000,000 pounds of tea from China to Great Britain in 1830 [54].

At present, tea popularity spreads across the world and becomes an international drink enjoyed by all people, especially middle-aged and older adults, as well as people with leisure and carefree moods. During the 1990s, Canadians began to purchase more tea instead of coffee and drank over 10 billion cups of tea annually. In 2015, $426 million dollar worth of tea was sold in grocery stores in Canada [55]. In Australia, adults over 45 years old drink more tea than coffee, and 22 million cups of tea are consumed each day [56]. In the UK, 165 million cups of tea are consumed each day, and Americans like tea as well and consumed about 3 billion gallons (11.3 billion liters) of it in 2010 [57].

#### Tea-planting dissemination

Since Chinese tea-drinking custom spread overseas and tea became a world’s recreational drink, other countries imported tea tree planting and cultivating techniques, tea processing and drinking methods, and tea ceremony (*Cha Dao* in Chinese) from China one after another. Figure 6C shows the dates when the first commercial planting of tea in other countries and regions started. Due to the lack of confidence on the wild tea in India, in 1833, East India Company collected 80,000 tea tree seeds from China and brought them to India [58]. Using the Chinese tea tree planting techniques, British launched a tea industry in Assam and started large-scale production and commercialization of the plant in India to bypass the China or Dutch monopoly on tea trade. Nowadays, tea plants derived either directly or indirectly from China have been introduced to more than 50 countries around the world for large scale commercial cultivation, and total planting area of tea has exceeded 4.1 million hectares (www.fao.org/faostat/). However, most countries and regions on the earth are not suitable for growing tea trees, because the temperature is too cold for the tea plants which are adapted to a hot and wet growing condition. Nowadays, tea plant breeding is a topic of great economic importance. The species of tea with resistance to colder weather and/or drought and those with high yields are preferably chosen in the tea planting industry.

At present, there are five tea-producing areas in 22 states with 13,000 tea gardens (beginning in the eighteenth century) in India, with Assam, Cachar, Darjeeling, Dehra Dun, Dooars, Kangra, Kerala, Manispur, Nilgiri, Terai, and Travancore being the main tea-producing areas [59]. Eight counties in Japan commercially grow tea (beginning in the twelfth century), with the main tea-producing counties being Shizuoka prefecture (20,300 hm^2^, accounting for 41.3% of the total area of tea cultivation), Kagoshima prefecture (8300 hm^2^, accounting for 16.7% of the total area of tea cultivation), and Mie (3,400 hm^2^, accounting for 6.8% of the total area of tea cultivation) [60]. In the continental USA, there are at least three tea-producing areas, i.e., Skagit Valley in Washington State, South Caroline and Alabama [61]. In some developing countries, tea is an important cash crop. Moreover, tea trees can help stop soil erosion and improve the environment, when they are planted on barrens. The tea planting industry has great potential. It is often regarded as an efficient way to advance the countries’ economic development and agricultural structure transformation. Due to the differences in the natural properties of tea, cacao, and coffee, it is impossible to find out how much we like them based on their planting area, annual output, quantum of international trade, or the price in the market. However, it is estimated that no more than 3 billion people worldwide drink cacao and coffee (Table 1). Among the three major drinks (tea, coffee and cocoa), tea is the most popular one in the world.

**Table 1:** In 2018, the top 10 countries of tea cultivation, output, import and export as well as tea country/per capita consumption, including coffee consumption, in the world

Country/tea cultivation area (hm^2^)	Country/tea output (ton)	Country/tea import (ton)	Country/tea export (ton)	Country/tea consumption (ton)	Country or region/per capita consumption	
					Tea (kg)	Coffee (kg)
China/3030,000	China/2,616,000	Pakistan/191,773	Kenya/474,862	China/1,911,000	Turkey/3.04	Finland/12.0
India/601,000	India/1,311,630	Russia/153,000	China/364,700	India/1,084,000	Libya/2.80	Norway/9.9
Kenya/234,000	Kenya/492,999	America/139,030	Sri Lanka/217,777	Turkey/246,000	Morocco/2.04	Iceland/9.0
Sri Lanka/303,000	Sri Lanka/303,843	Britain/107,862	India/245,100	Pakistan/192,000	Ireland/1.79	Denmark/8.7
Vietnam/134,000	Turkey/252,000	CIS/90,000	Vietnam/136,000	Russia/162,000	Britain/1.62	Netherlands/8.4
Indonesia/115,000	Vietnam/168,000	Egypt/82,000	Argentina/78,000	America/120,000	Hong Kong/1.52	Sweden/8.2
Burma/80,000	Indonesia/131,000	Morocco/73,000	Uganda/50,000	Britain/107,000	China/1.48	Switzerland/7.9
Turkey/77,000	Bangladesh/82,134	Iran/63,600	Indonesia/49, 030	Japan/106,000	Afghan/1.37	Belgium/6.8
Bangladesh/59,000	Argentina/80,000	Dubai/63,000	Malawi/34,816	Indonesia/103,000	Sri Lanka/1.35	Canada/6.5
Uganda/45,000	Japan/79,000	Iraq/40,600	Tanzania/26,700	Egypt/94,000	Katar/1.34	Luxembourg/6.5

CIS (Commonwealth of Independent States)

Data from https://www.tanmizhi.com/html/4991.html, http://data.chinabaogao.com/shipin/2020/091K154362020.html, https://www.tanmizhi.com/html/4991.html, and https://www.sohu.com/a/321377588_482413;

## Tea art, a symbol of Chinese culture

**Fig. 7:**
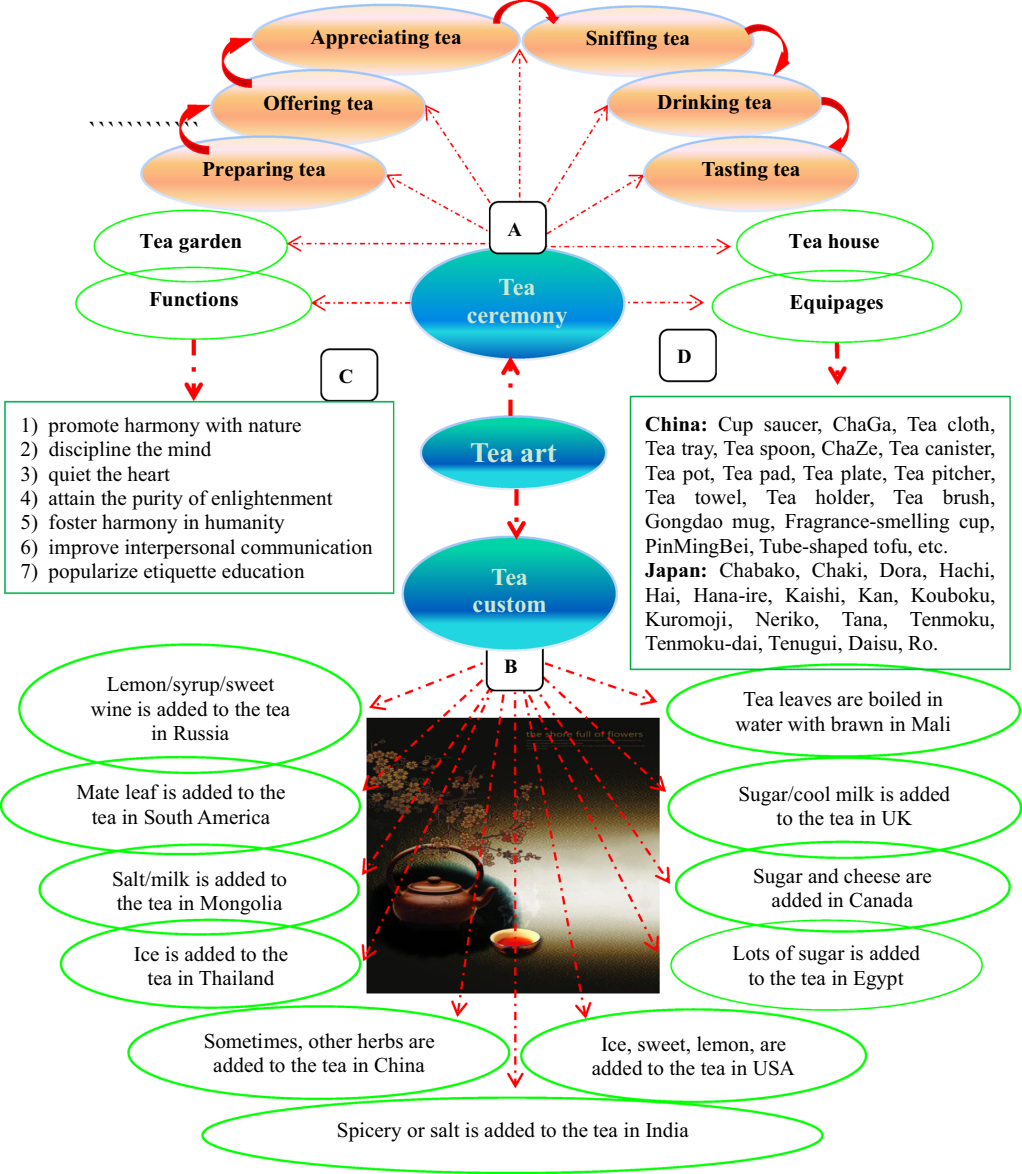
Tea culture/art: tea ceremony (**A**) and tea custom (**B**)

**Fig. 8:**
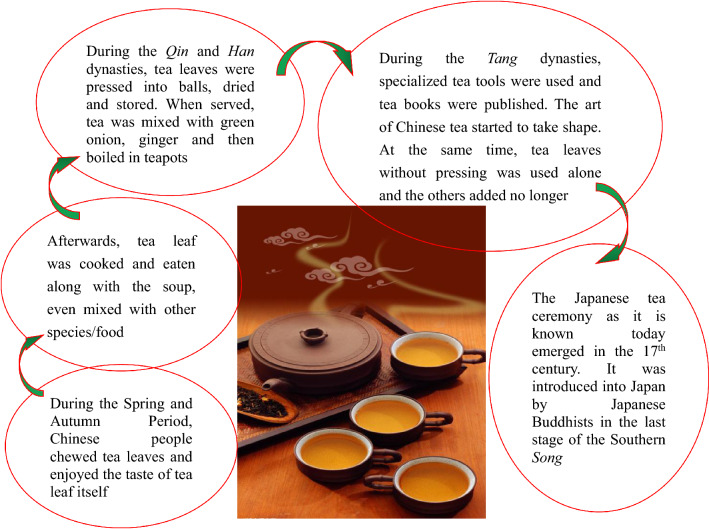
Development of tea custom/ceremony

Tea art, an important cultural item and symbol nowadays, is a simple (tea custom) or complex (tea ceremony) performing art with combined elements including people, tea, water, utensils, skills and the environment, which expresses the spiritual pursuit of tea markers and drinkers. The importance of tea art depends on the positive effects of tea on human physiological, mental and emotional health, and social etiquettes that affect interpersonal relationship. We consider that tea art includes both tea ceremony (Fig. 7A) and tea custom (Fig. 7B) [62]. The development of tea art is shown in Fig. 8 [63].

### Tea custom

Tea custom refers to the habit of tea-making and tea-drinking among a specific region, society, or colony, which may be simple or complex based on the tea-makers’ and tea-drinkers’ eating habits, food culture and life-style. In China, tea art began in the *Tang* dynasty and developed during the *Song* dynasty [64]. Afterwards, tea was introduced into other parts of the world, and drinking tea has become an elegant style, the best way to greet guests, and a habit and custom in many countries and regions. Someone may feel dejection when there is a lack of tea in his daily life. To some extent, there is hope when there is tea. For instance, in Britain, there are early tea, breakfast tea, morning tea, lunch tea, middy tea, afternoon tea (low tea), before dinner tea (high tea), after dinner tea, and night tea [65].

Tea-drinking customs are significantly different among different peoples with different life patterns, dietary habits, religions and cultures. For example, since there are Confucians, Taoist, and Buddhist, as well as people without any religion in China, Chinese tea arts include Confucianism tea, Taoism tea, Buddhism tea, and vulgar tea (俗茶 in Chinese; “tea of the public”), which conform to the corresponding religious morals and behavior standards. Taoists regards drinking tea as self-cultivation and for keeping the oneness of soul and body; Buddhists consider that drinking tea is useful for the deep understanding of *Zen*; and tea and drinking tea are regarded as a way of hospitality and an embodiment of the humanistic quality by Confucians.

Russia is a nation where people like to drink liquor, and thereby Russians sometimes make tea with liquor. In China, Chinese tea is sometimes made with other herbal medicine based on the principles of traditional Chinese medicine (TCM) and CHM. Hot tea is most popular in China. Due to the unique cultural traditions and the way of hospitality, British people particularly enjoy black tea. When Americans consume tea, they pay attentions to the color, rather than the form of tea, and many of them like to drink ice tea. Therefore, tea bags, instant tea and tea powder are sold in USA’s markets. The reason for this is that America is a multicultural country with a quick pace of life, and the people emphasize on the actual state of substances. In India, because of the damp climate, tea leaves are usually boiled together with spicery such as cardamom, cinnamon, ginger, cloves, and even black pepper and star anise.

### Tea ceremony

Tea ceremony is a kind of dietary culture, and it may meet the needs of the human spirit (enjoyment) and body (supplement of water and some bio-activators). It usual takes place in such occasions as wedding days, birth days, parties, traditional festivals, and some other significant events. In fact, tea ceremony is not only for drinking tea but also for learning about traditional etiquette cultures. Moreover, it creates a link among other arts such as landscape, architecture, poetry, calligraphy, pottery and cuisine [66]. Therefore, we could say that tea ceremony improves our aesthetic standards and edifies our sentiments (thoughts and feelings).

#### The forms of tea ceremony

China is an etiquette nation and a state of ceremonies (礼仪之邦). The word “ceremony” (*L*i, 礼) is the basis or gene of Chinese culture and the first syllable of Chinese civilization, as it says “Of all things, courtesy comes first” (万事礼为先). Even fighting with each other, the two opponents are still supposed to be refined and courteous (彬彬有礼), and shall try peaceful means before resorting to force (先礼后兵). Ceremony is not a hollow word in China, rather, there are many medias and carries. Tea is one of them, which is named as tea ceremony, tea culture, tea way, Teaism, tea art, or “*Cha Dao*” (茶道) in Chinese. The word *Cha Dao* (tea ceremony) first appeared in a poem written by a monk poet *Jiao Ran* (730–799) in the *Tang* dynasty*.* Like religious rituals, tea ceremony, an etiquette based on natural plant leaves, has fixed formal forms. The criteria include the following: how to make/prepare tea, serve/offer tea, appreciate tea, sniff tea, savor/drink tea, and taste tea in the order of performance, as well as how to accurately use tea-related equipages. There are 29 kinds of tea equipages and 36 kinds of normative actions/behaviors in Chinese tea ceremony, and 17 kinds of tea equipages in Japanese tea ceremony [67–70]. Tea ceremony is the elegance of tea-drinking.

**Fig. 9:**
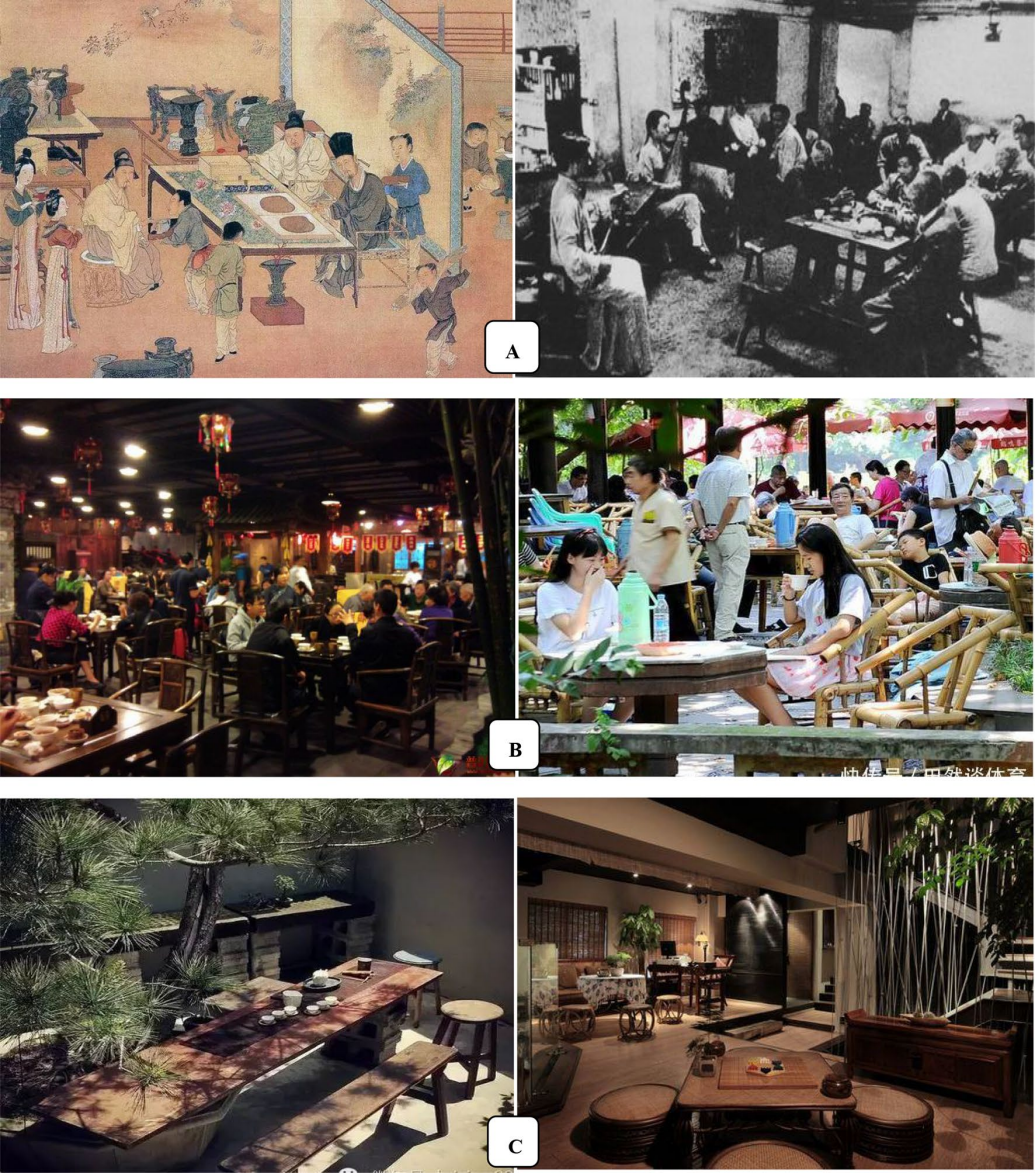
Tea house (茶馆) in China. **A** Old tea house; **B** popular tea house nowadays; **C** high-end tea house nowadays. Data from http://www.360doc.com/content/19/0429/16/6795100_832319897.shtml (**A**) and https://image.so.com/i?src=360pic_strong&z=1&i=0&cmg=15484592.2804341344203044000.1611559700524.4019&q=%E4%B8%AD%E5%9B%BD%E8%8C%B6%E9%A6%86 (**B** and **C**)

Some 242 kinds of tea-packing equipages are available in China (Fig. 7D). Tea making involves tea, water and tea-china selection, tea-making techniques, and the environmental refinement which includes a tea house decorated with traditional paintings, calligraphy and furniture and a tea garden as a setting for the tea ceremony. In Japan, there are ten famous tea ceremony houses, including the Kodaiji temple Ihoan (Iho-an) tea hut, Katsura Rikyu Imperial Villa Shokin-tei, Katsura Rikyu Imperial Villa Geppa-ro, Joan Tea Pavilion, Jikouin Tea Pavilion, Choushukaku Residence in Sankei Garden, Isuitei tea pavilion, Kasumidoko-seki tea room, Sa-an tea house, and Kan'in-no-seki tea room [71]. Nowadays, when meeting important people or making a major deal, expensively-decorated tea houses are usually selected, besides restaurants and hotels. However, ordinary public can play games and chat with friends in simple tea houses which may not look elegant. In China, ancient scholars enjoyed themselves by savoring tea in bamboo groves or in the moonlight. Different tea houses can meet different needs of individuals (Fig. 9).

Upon entering a tea ceremony setting, what are the appropriate behaviors and the correct tea-making manners we should abide? Tea ceremony has been used as a sacred and meditative ritual in Japan throughout the ages. From marking tea to offering tea and drinking tea, all aspects of the ceremony demand your mindfulness and care. The mindfulness even begins in the cultivation and processing of tea. Geishas in Japan are great masters of tea art. However, Geisha tea art is too complex to be handled by most people without much knowledge of tea ceremony [72]. Moreover, most people in the world do not have the opportunity of getting the enjoyment of formal tea ceremony. In the Chinese society, it is the simple tea ceremony that the younger generations use to show their respects to the older generations or guests by offering a cup of tea with both hands, and sometimes, people make apologies to others by pouring tea for them [73].

#### The functions of tea ceremony

Tea ceremony originated more than 1000 years ago in China, but it is still one of the most significant events in modern society. Nowadays, it has been spread all over the world, including Japan (beginning in the Muromachi period), Korea (beginning in the Silla era) and Britain (beginning in the Victorian era). As we know that the Japanese tea ceremony, also called *Chanoyu, Sado*, or *Ocha* in Japan, with its own special features, is quite elegant. In comparison, the ordinary Chinese tea art pays more attentions to the tea itself, such as tea tastes, tea smells, and the choice of tea varieties. Japanese tea art emphasizes the expression of tea ceremony and enjoyment of the tea-drinking procedure. It is a highly structured ritual centered on the preparation, service, and consumption of tea. Matcha, a special powdered green tea with high concentrations of epigallocatechin gallate (EGCG), is usually used in Japanese tea ceremony [74]. Some tea ceremonies in China are as elegant as those in Japan, although most tea drinkers in China often like to judge whether or not the tea they are drinking is famous and authentic.

The philosophy of Japanese tea ceremony is expressed as Wa (harmony, 和), Kei (respect, 敬), Sei (purity, 清), and Jaku (tranquility, 寂) [75]. Chinese moral emphasizes on *Lian/Qing* (廉/清, righteousness), *Qin* (俭, thrift), *He/Rong* (和/融, harmony), *Jing* (敬, respect), *Li/Li* (理/礼, truth/courtesy), *Xing* (性, mood), *Chun* (纯, purity), *Lun* (伦, ethics), *Ji* (寂, silence), Jing (静, quietness), *Le* (乐, happiness), *Mei* (美, beauty), and *Jian* (健, health) [76]. Chinese tea ceremony takes the “Four Truths” (四谛) as its general principle, namely, peace (和), tranquility (静), happiness (怡) and truth (真). Today, most of us are living in a fast-paced society and hardly have time to slow down long enough to appreciate natural beauty and the beauty of life and human life, and to enjoy our soul of purity and peace. At present, the human being seems to be coerced by some demon (self-interests) and is running in the direction of death, with the destruction of ecological environment and the fragmentation of the society. The tea ceremony can give us a chance to achieve the scene of harmony, peace, respect, purity, and tranquility in our minds through serving tea heartily, receiving it with gratitude, and offering it to others.

All in all, tea ceremony is a very professional work, involving the tea set and the moves and acts of the tea offering person/server and drinkers, and is full of artistic and cultural breath and the show of personal accomplishment and elegance of the participators. In fact, tea ceremony is a kind of mental self-complacence, soul enjoyment, and even religion and metaphysics, which is far beyond the biological efficacy of tea liquor itself (Fig. 7C). Generally, participators of tea ceremonies use a small cup (just large enough to hold about two small mouthfuls of tea) to drink teat, and “savor” is the word used to describe this behavior. On the other hand, like a cow drinking water, ordinary people usually use a big cup of water or drink to quench their thirst, so “drink” is used to illustrate this action. In fact, there is no scientific and exact definition of tea ceremony or tea moral, and people need to comprehend it through their own existence. However, tea ceremony does not equal to drinking tea. At the tea ceremony tea is often regarded as a precious and gracious beverage. It is believed that a tea-drinking process is a spiritual enjoyment, an art, a means of cultivating the moral character and nourishing the mind, and a kind of etiquette education for teenagers. In addition, it is also very important to appreciate tea itself during the tea ceremony, to enjoy the beauty of the tea name, tea shape, tea color, tea fragrant, and tea taste [77].

## The types of Chinese tea

**Fig. 10:**
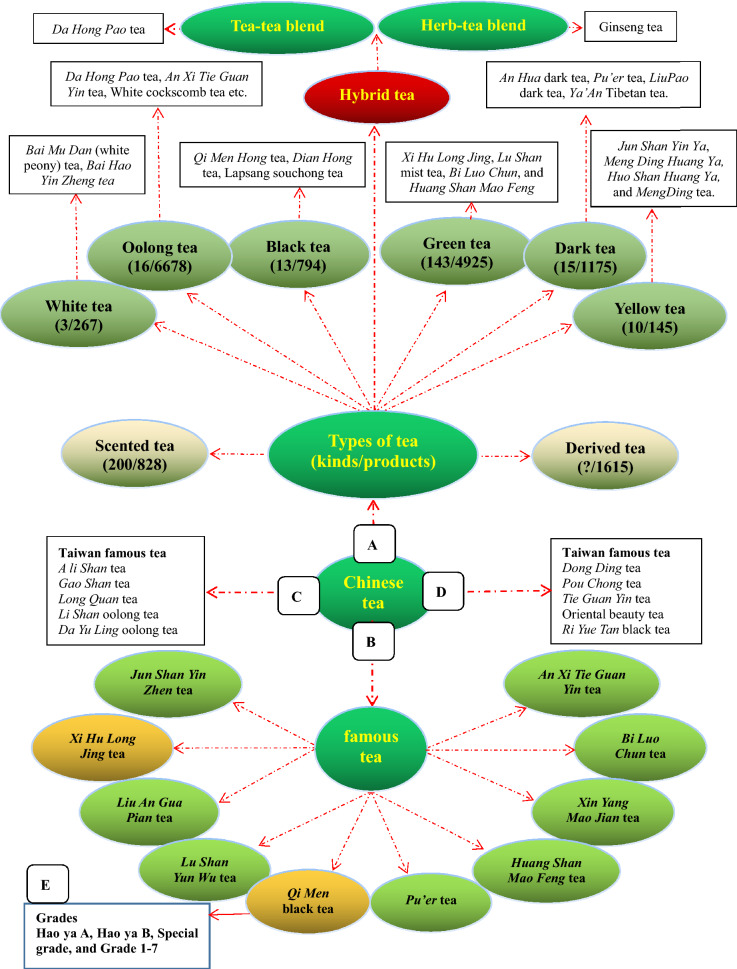
Tea forms (**A**), famous teas (**B**, **C**, **D**), and *Qi Men* black tea grade (**E**)

Different types of Chinese tea and Chinese hybrid tea currently in the market are shown in Fig. 10. There are more than 3000 kinds of tea in the world, but no accurate data are available. Records of 16,027 kinds of Chinese tea and tea-related products can be found in the internet. Some 10,000 tea-related products are shown in Taobao (www.taobao.com), a well-known Chinese e-trade platform. In China, tea is generally classified into 6 major types, distinguished mainly by the methods of production (Fig. 10A), according to such parameters as the degree of tea leaf fermentation, tea tree growing conditions, horticultural methods, tea-manufacturing arts, and the time of tea leaf harvest, etc. [78–80]. Ten famous types of tea in mainland China determined by the Panama-Pacific International Exposition held in San Francisco in 1915 are shown in Fig. 10B, and ten famous kinds of tea in the Chinese *Tai Wan* are shown in Fig. 10C and D.

### Single-tea types

There are over a thousand different varieties of tea, but the most common ones in China can be classified as green tea, black tea, oolong tea, dark tea, white tea and yellow tea.

#### Green tea (*Lu Cha*, with a history of approximately 3000 years)

Green tea, the only category of tea that is un-oxidized (non-fermented tea), is made from fresh leaves of the *Camellia sinesis* plant. It is processed using either artisanal methods (sun drying or basket/charcoal/pan frying) or modern methods (oven drying, tumbling or steaming). Green tea gets its name from its yellowish-green liquor and green leaves which are not fermented. Green tea usually has a faint scent with a slightly bitter and astringent taste. In China, there are 143 kinds of green teas, but the famous representatives are *Xi Hu Long Jing* tea, *Lu Shan* mist tea, *Bi Luo Chun* tea, and *Huang Shan Mao Feng* tea. The annual output of green tea accounts for 22% of the total tea output in the world. In 2015, the output of Chinese green tea was 1,438,000 tons and the export volume reached 272,000 tons. In 2019, the output of green tea was 1,772,900 tons, accounting for 63.47% of total tea output in China. In 2020, green tea export volume reached 293,400 tons, accounting for 84.1% of China’s total tea export volume.

#### Black tea (*Hong Cha*, with a history of approximately 400-years)

Black tea, the nearly completely fermented tea with a strong flavor and dark color, which is commonly known as “red tea” (red color of the tea liquor) in China, is developed on the basis of green tea. The production procedures of Chinese black tea include withering, rolling, fermenting (80–90% fermentation) and drying. The tea liquor has a dark color and strong flavor which may be described as floral honey fragrance, but different tea origins produce different black tea flavor profiles. *Qi Men Hong* black tea of *Qi Men* county in *An Hui* province, *Yun Nan Dian Hong* tea from the southern and southwestern areas of *Yun Nan* province, and *Lapsang Souchong* tea from the *Wu Yi* region of *Fu Jian* province are the famous representatives of black tea. Top grade black teas are made from the tea tree leaves picked in the summer season. In 2015, the output of Chinese black tea was 258,000 tons and the export volume reached 28,000 tons. In 2019, the output of black tea was 307,200 tons, accounting for 11% of the total tea output in China. In 2020, black tea export volume reached 28,800 tons, accounting for 8.3% of China’s total tea export volume.

#### Oolong tea (*Wu Long Cha,* with a history of approximately 290 years)

Oolong tea, a class of half-fermented (30–60% fermentation) tea, is in-between black tea and green tea in color and taste, combining the good qualities of green tea and black tea. Its taste is neither the rosy and sweet aroma of black tea nor the grassy-vegetal notes of green tea. It is commonly brewed to be strong with a bitterness, leaving a sweet taste after drinking. In China, there are 10 famous oolong tea such as *Da Hong Pao* tea from the *Wu Yi* mountain of *Fu Jian* province, *An Xi Ti Kuan Yin* tea of *An Xi* county in *Fu Jia*n province, White cockscomb tea (*Bai Ji Guan* tea) from the *Wu Yi* mountain of *Fu Jian* province, etc. In 2015, the output of Chinese oolong tea was 258,900 tons and export volume reached 15,000 tons; in 2019, the output of oolong tea was 275,800 tons, accounting for 9.87% of the total tea output in China. In 2020, oolong tea export volume reached 16,900 tons, accounting for 4.9% of China’s total tea export volume.

#### Dark tea (*Hei Cha*, with a history of approximately 3,300 years)

Dark tea is post-fermented (100% fermentation) tea that has undergone further “aging” through auto-oxidation, fermentation and other enzymatic reactions after being made and before being sold. Dark tea is widely welcomed by the minorities of China, especially Tibetan and Mongolian people. During the fermenting procedure, tea is darken, giving this tea category of its name. The tea liquor has a strong aroma with a thick and robust flavor, which leaves a long lasting aftertaste. This type of tea may be loosely packaged or compressed into cakes/bricks/bowls for storage and shipping. The famous dark tea includes *An Hua* dark tea from *An Hua* county in *Hu Nan* province, *Pu'er* tea of *Yun Nan* province, *Liu Pao* dark tea of *Guang Xi* province, and *Ya An* Tibetan tea of *Ya An* city in *Si Chuan* province. In 2015, the output of Chinese dark tea was 297,100 tons and export volume reached 156,300 tons. In 2019, the output of dark tea was 378,100 tons, accounting for 13.54% of the total tea output in China. Dark tea was the earliest tea exported from China through the Silk Road during the *Western Han* dynasty [81]*.*

#### Yellow tea (*Huang Cha*, with a history of approximately 2000 years)

Yellow tea, like dark tea, is a non-enzymatically oxidized, slightly fermented (10–20% fermentation) tea. During the processing, the color of tea leaves change from green to yellow, so that this tea is called yellow tea. It includes three types: (1) *Huang Ya* tea (“yellow-bud tea”) represented by *Jun Shan Yin Ya* (“silver bud of the *Jun Shan* mountain”) made in *Hu Nan* province, *Meng Ding Huang Ya* (“yellow bud of *Meng Ding*”) produced in *Si Chuan* province, and *Huo Nei Ya* from *An Hui* province; (2) *Huang Xiao* tea (“yellow small tea”) represented by *Huo Shan Huang Ya* (“yellow bud of the *Huo* mountain”) of *An Hui* province and *Meng Ding* tea of *Si Chuan* province; and (3) *Huang Da* tea (“yellow big tea”) represented by *Huo Shan Huang Da* tea of *An Hui* province and *Da Ye Qing* tea (“big leaf light tea”) of *Guang Dong* province. Yellow-bud tea, is a rare, top grade yellow tea, which was/is sent as tributes to emperors/honored ones. The tea liquor has a fresh and mellow taste, and the liquor color is yellowish bright and shining, with a clean and fresh aroma. In 2015, the output of Chinese yellow tea was 3472 tons; and in 2019, the output of Chinese yellow tea was 9700 tons.

#### White tea (*Bai Cha*, with a history of approximately 900 years)

**Fig. 11:**
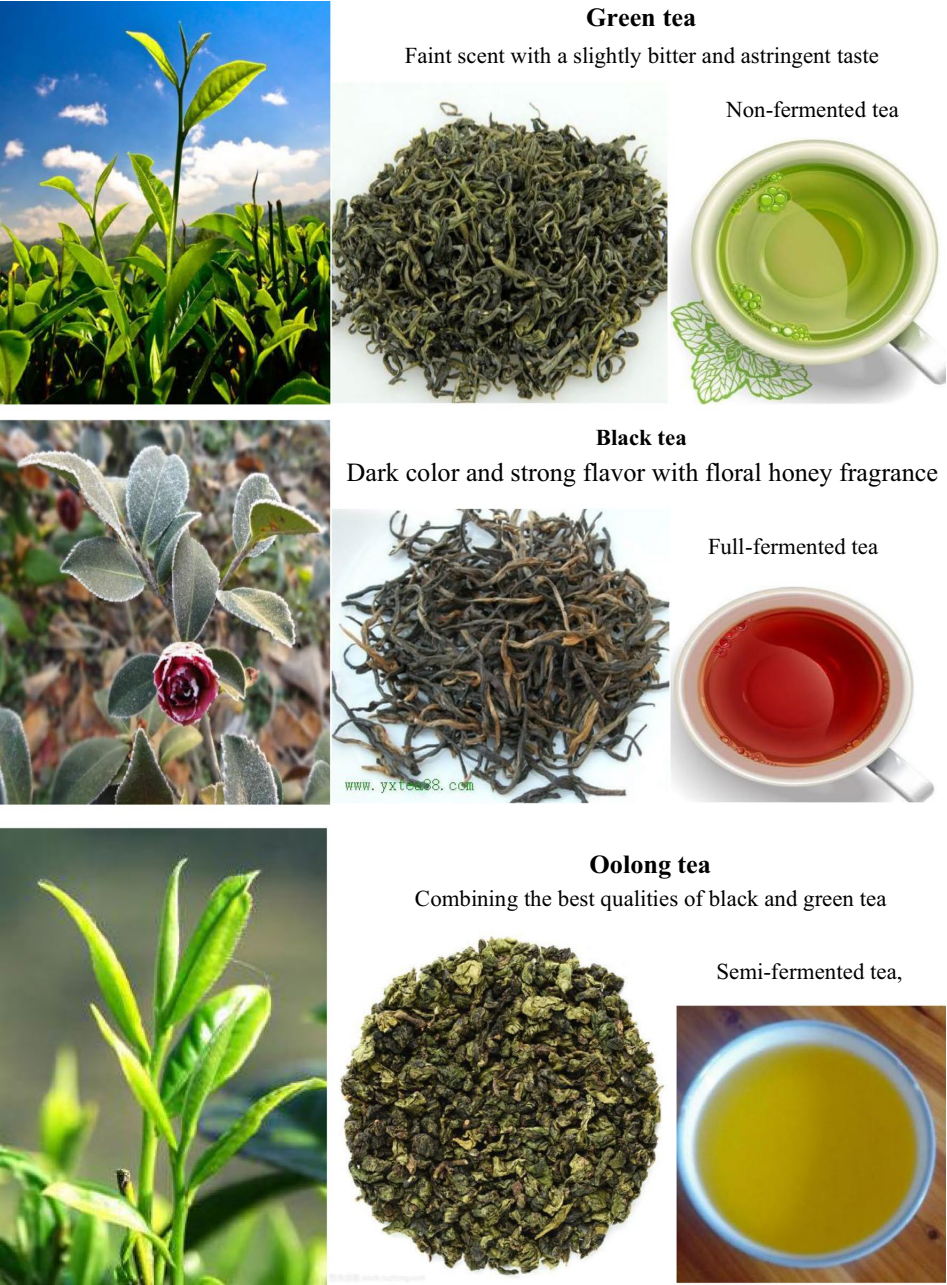

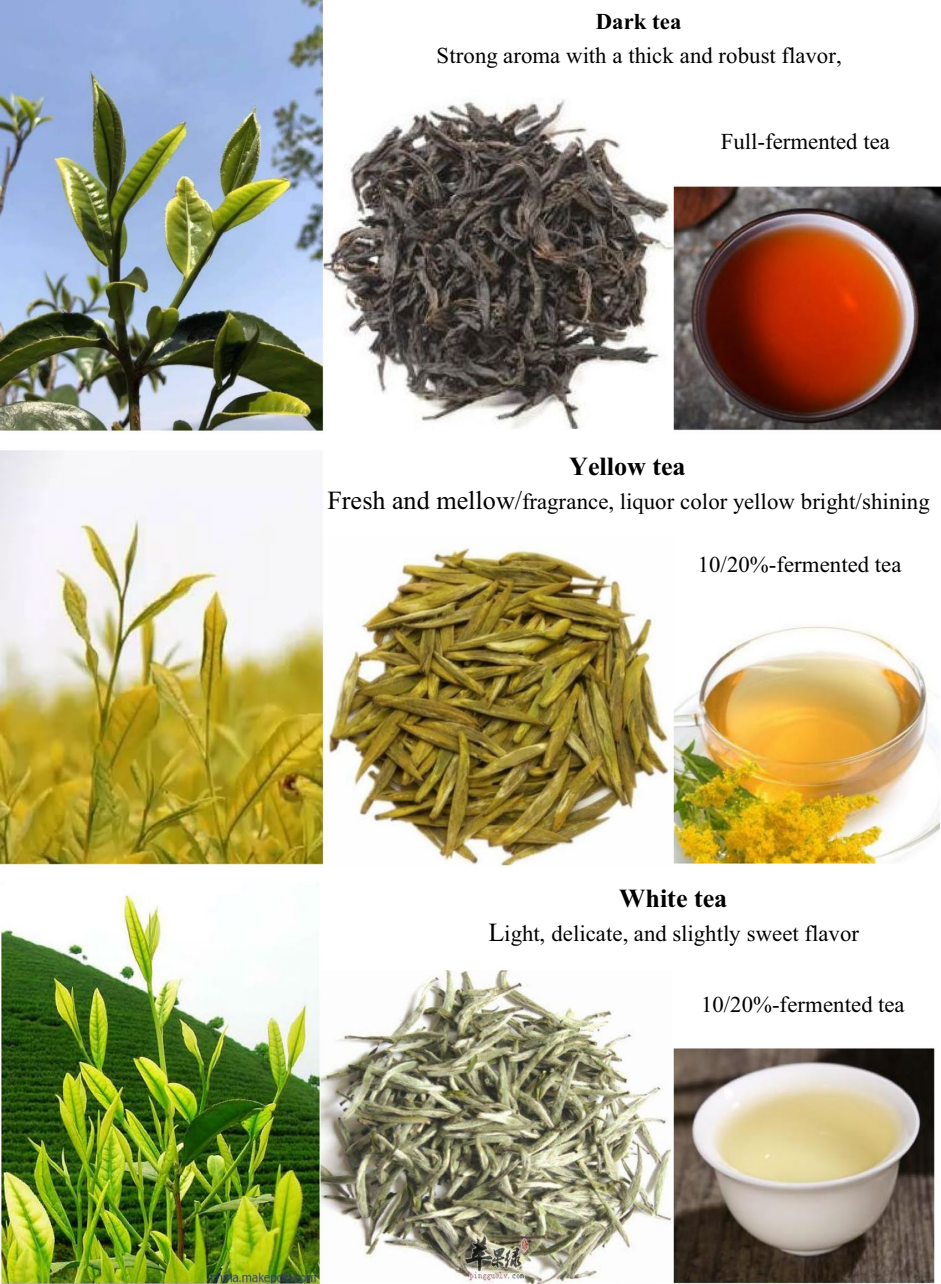
Six major kinds of tea in China. Data from https://image.so.com/i?src=360pic_strong&z=1&i=0&cmg=15484592.1427114627743071700.1627690750402.8748&q=%E4%B8%AD%E5%9B%BD%E7%BB%BF%E8%8C%B6%20%E5%9B%BE%E7%89%87, https://image.so.com/i?q=%E4%B8%AD%E5%9B%BD%E7%BA%A2%E8%8C%B6+%E5%9B%BE%E7%89%87&src=srp, https://image.so.com/i?q=%E4%B8%AD%E5%9B%BD%E4%B9%8C%E9%BE%99%E8%8C%B6+%E5%9B%BE%E7%89%87&src=srp, https://image.so.com/i?q=%E4%B8%AD%E5%9B%BD%E9%BB%91%E8%8C%B6+%E5%9B%BE%E7%89%87&src=srp, https://image.so.com/i?q=%E4%B8%AD%E5%9B%BD%E9%BB%84%E8%8C%B6+%E5%9B%BE%E7%89%87&src=srp, and https://image.so.com/i?q=%E4%B8%AD%E5%9B%BD%E7%99%BD%E8%8C%B6+%E5%9B%BE%E7%89%87&src=srp

White tea, also known as “silver tips”, is highly prized, with prices significantly higher than other types of tea. White tea is a kind of slightly fermented (10–20% fermentation) tea with a white color. Good-quality white tea is made using only the young tea leaves with lots of fine hairs (silvery white buds). The making procedure of white tea is separated into withering and drying, and it does not require panning, rolling or shaking. Therefore, white tea contains more nutrients than other teas, because of its minimal processing. White tea has a light, delicate, and slightly sweet flavor. *Bai Hao Yin Zheng* and *Bai Mudan* (white peony) are the most famous white tea [82]. In 2015, the output of Chinese white tea was 22,000 tons and export volume was 4400 tons (Fig. 11).

Each of the six types of teas described above can be further divided into several subclasses based on tea-processing procedures and their qualities.

### Hybrid-tea type

Tea is classified as *Camellia sinensis* tea and non-*Camellia sinensis* tea, such as herbal tea. Hybrid tea or mixed tea refers to the fusion of tea with another tea or other substances, which is made by combining different varieties of teas/herbs to produce a special taste and different health effects.

#### Scented tea

**Fig. 12:**
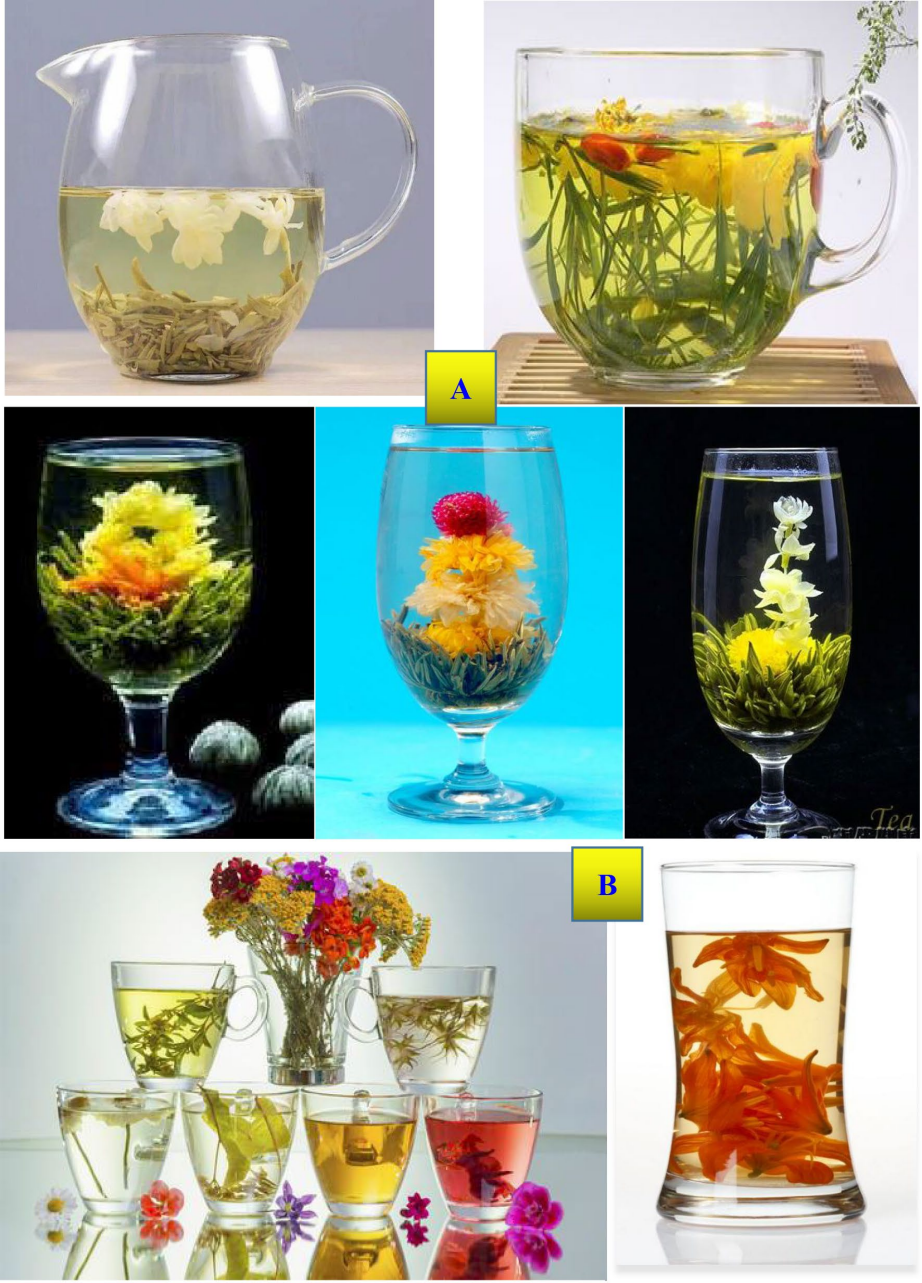
Scented tea with tea leaf (**A**) and scented tea without tea leaf (**B**). Data from https://image.so.com/i?src=360pic_strong&z=1&i=0&cmg=15484592.1427114627743071700.1627690750402.8748&q=%E8%8A%B1%E8%8C%B6%E5%9B%BE%E7%89%87%E5%A4%A7%E5%85%A8%E5%A4%A7%E5%9B%BE

To make scented tea (with more than 1000 years of history), green tea is scented with aroma from fresh sweet-smelling flowers such as Arabian jasmine flower, magnolia flower, *Osmanthus fragrans Lour*., peony, rose, peach blossom, calendula, etc., through traditional or modern scientific and technological processing. Afterwards, flowers are removed from the tea or left in the tea. Scented tea in the market includes: (1) tea is only scented with plant flower petals through an elaborate process; (2) tea is made by mixing green tea with plant flowers; (3) plant flower is dried like tea (Fig. 12). In 2015, the export volume of Chinese scented tea was 6045 tons. In 2020, the output of scented tea was 6100 tons, accounting for 1.8% of the total tea output in China.

#### Blend tea

Blend tea (mixed tea) is a very complicated concept. Tea-tea blend, herb-/flower-tea blend, and even food-tea blend are also popular in the world. For example, British breakfast tea includes Assam tea, Ceylon tea, and Paraguay tea according to a certain blend composition. In China, *Da Hong Pao* tea is blend tea with a mixture of four varieties of tea plant leaves, and *Lao Ban Zhang* tea is constituted by several varieties of tea plant leaves. A Japanese food is comprised of green tea mixed with brown rice. The directory of THEODOR tea bar in France has a total of 100 hybrid teas mixed with 450 kinds of tea [83]. These mixed teas have a balanced and fixed taste and nearly the same quality. Additionally, there are many herb-/flower-tea blend products that can restore health and vitality in China’s market. It has been demonstrated that various types of teas or herbs/plant flowers, and even medicinal drug combinations, act synergistically to exhibit health benefits to humans, improve consumer acceptance and economic value, e.g., increase in the effectiveness of teas, herbs and medicines.

### Derived-tea type

Derived tea means tea-related drinks or drinks named tea which are not derived from tea leaves. It includes four classes as follows [84].

1. Extracts of tea or fresh tea leaves, which lost the morphology of tea leaves and the feelings of tea culture and art, such as tea polyphenolic extracts obtained from green tea [85].
2. Tea-like drinks made from the extracts/powder of herbal medicine by a modern scientific process under strict quality control, such as American ginseng tea made using American ginseng (*Panax quinquefolius*) roots [86].
3. Dried herbs/plants not belonging to the species of theaceae plants are used in the form of tea as an infusion (adding hot water) or a decoction (herbs and water are boiled together), i.e., “tea substitute” (代茶饮) or “tea out of tea”, such as Chinese ginseng tea made using Chinese ginseng (Panax ginseng) leaves or flowers [87, 88], gynostemma tea made from *gynostemma pentaphylla* (herbal medicine), and Paraguay tea produced using the leaves of *Ilex paraguariensis* plant, Java tea (*Orthosiphon stamineus*) with diuretic and antioxidant activities [89, 90], Yareta tea (*Azorella compacta*) with health-promoting property, a moss growing in the Andes highland [91], and *baccharis trimera* popularly consumed as tea in Candiota (Brazil) [92]. In China’s market, derived teas that are not real tea derived from the plant *Camellia sinensis (L.) O. Ktze* include the fellowing: *Gynostemma pentaphyllam* tea, *Eucommia ulmoides* tea, pine needle tea, *Herba plantaginis* tea, *Apocynum venetum* tea, chrysanthemum tea, boat-fruited sterculia tea, *Lonicera japonica* tea, red sage tea, osmanthus tea, acanthopanax root tea, senna leaf tea, persimmon leaf tea, green bean tea, genmaicha tea, rhynchophylla tea, crispy rice tea, hawk tea, ginger tea, red date tea, corn whisker tea, and bamboo leaf tea, etc.
4. Although Chinese teas are divided into green tea, yellow tea, white tea, oolong tea, black tea, dark tea, and hybrid tea according to their color or production process, they may also be classed as spring tea (harvested before late March to mid-May), summer tea (harvested from early May to early July), autumn tea (harvested after the middle of August), and winter tea (harvested in late October) based on the tea-leaf picking seasons. Generally, spring tea is rich in vitamins and amino acids, and has a fresh and pleasant taste and very good health functions. Because the contents of anthocyanin, caffeine and tea polyphenols are higher than those of spring tea, summer tea has a more bitter taste. The aroma is not intense and the taste is thin for autumn tea. Winter tea is very rare in the market. Additionally, depending on the growing environment, Chinese tea can be classified into *Ping Di* (flat area) tea and *Gao Shan* (high mountain) tea.

## Top-grade black teas worldwide

Black tea, known as red tea (*Hong Cha* in Chinese) in China, has become the most popular tea in the world, because of its heavy and yet smooth taste. It accounts for over 90% of all tea sold in the western countries and 66% (2010) of all tea output in the world. In 2018, 55,014 tons of Chinese black tea were exported, accounting for 4.07% of the global black tea export (1,350,885 tons) [93].

### *Qi Men* black tea

Today, there are 13 types of black tea and 794 related products in the Chinese tea market. Lapsang Souchong tea, the first black tea in history born in the *Wu Yi* mountain range of China in 1568 and emerged in Europe in 1604, is the ancestor of modern black teas. *Qi Men* (also named Keemun) black tea was first manufactured in 1875, having 146 years of production history. It has been awarded many prizes in international and domestic contests, including the Gold Prize of the International Fair in 1915, 1980, 1985, 1990 and 1995; the Gold Prize of the 26th World High Quality Food in 1987; and the medal of China Tourism new product “Pegasus Gold Award” in 1992, etc. In recent years, more and more tea connoisseurs take to Assams and Ceylon black teas, but the premium quality *Qi Men* tea still remains the title of “The king of black teas” [94].

*Qi Men* black tea is famous for its unique strong floral honey-like flavor and fragrance and its thickness and mellowness. After drinking the tea liquor, you will experience a long after sweetness and aroma in your breathe. Adding some milk or honey, you will get different enjoyment for its taste. High-quality *Qi Men* black tea is inseparable from the *Qi Men* region with the natural ecological conditions. *Qi Men* country is located at the southern tip of *An Hui* province of China, with an area of 2257 km^2^. In this region, the mountain area accounts for 90% of the total area, and the average height is 600 m above sea level. About 80% of the tea is distributed at an altitude of 100–350 m in the canyon area, and the forest coverage rate is as high as 88%. The gardens (with a total area of more than 10,000 km^2^) for planting *Qi Men* black tea are scattered over the mountainous area with fertile red/yellow soil covered by forest and fog, which can block direct sun light on tea leaves. The *Qi Men* region has an average of about 50 sunny days, 170 cloudy days, and 150 misty/rainy days a year. In addition, there is sufficient rainfall (average 1,726 mm annual rainfall), high humidity (80% in spring and summer), relatively low temperatures (average annual temperature of 18 °C; average 26 °C in July and average 4 °C in January), and a big temperature difference between day and night in this area [95]. These tea-growing conditions make *Qi Men* tea rich in theanine, a free amino acid only found in tea leaves. Theanine is responsible for making the tea with a long-lasting aroma and a non-astringent but mellow and sweet taste [96]. If tea leaves are exposed to sunlight, theanine will convert into catechin, a kind of polyphenols [97]. Besides, this tea contains a vast amount of myrcenal, an essential oil, and geraniol whose content is 40–100 times higher than that of ordinary tea [98]. These compounds make *Qi Men* tea smells like a dried black rose. The tea tree leaves for making *Qi Men* tea are usually plucked between April and July. There are 12 steps of drinking *Qi Men* black tea during the tea art performance.

The output of *Qi Men* black tea amounted to 2409 tons in 2006, 4000 tons in 2010, and 5000 tons in 2014, among which the quantity of Hao ya A and Hao ya B were only 2 and 10 tons, respectively, in 2006. At present, the planting area of *Qi Men* black tea reaches 9700 km^2^, including 1099 km^2^ of organic tea gardens certified by European Union BCS. There are 114 enterprises of *Qi Men* black tea manufacturing in China [99, 100].

### Darjeeling black tea

**Fig. 13:**
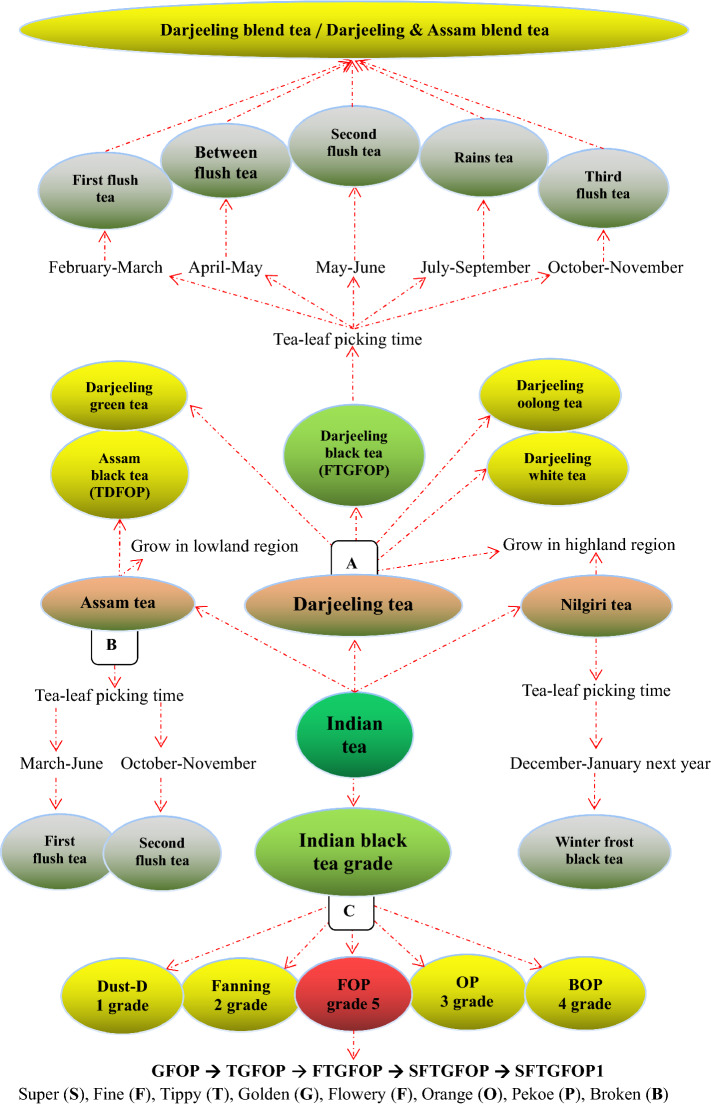
Indian black tea and Indian teas

Darjeeling teas in India are shown in Fig. 13A. Because the tea industry in India was established and developed by the British who have heavy tastes, annual production of more than 950,000 tons of Indian tea is almost entirely black tea to meet the western people’s tastes [101]. In the Alibaba website (Alibaba.com), 2379 messages about Indian tea, including 78 products of Darjeeling black tea, were found. Tea planting in the Darjeeling district of Indian West Bengal began in 1841, and the Darjeeling Tea Company was established in 1856. Darjeeling tea, the first product from India to receive the GI tag, is normally made from the small-leaved Chinese variety of *var*. *sinensis*, rather than the large-leaved Assam plant (*C. sinensis var. assamica*). At present, Darjeeling oolong, green and white teas are also found in the tea market, but black tea, with annual output of 8000–11,000 tons, is the main product of Darjeeling tea with an annual production of 16,535 tons [102]. All types of Darjeeling teas are immensely fragrant, with perfume-like, delicate and sweet smells, and are particularly favored as breakfast teas.

Darjeeling town/city, a municipality in the Indian state of West Bengal that grows Darjeeling tea, is located in the beautiful hillside of the Himalaya mountain at an altitude of 7000 feet above sea level. The best Darjeeling teas are produced at an elevation of 2500–5000 feet. Darjeeling has a climate with wet summers caused by monsoon rains. The sky is usually enveloped in clouds, the weather is pleasantly cool (monthly mean temperatures ranging from 6 to 18 °C), and the rainfall is ample (309.2 cm of average annual rainfall) [103]. When grown, brewed and processed in this area, the tea has a distinctive, naturally occurring aroma and taste, with light tea liquor, and the infused leaves also have a distinctive fragrance. These make Darjeeling black tea become one of the best quality teas in the world. Today, there are about 102 tea gardens covering a total area of over 17,500 km^2^ in the Darjeeling region, most of which are legacy gardens, being home to tea plants that are more than 100 years old. The famous gardens include Badamtam, Balasun, Bannockbum, Castleton, Gielle, Glenburn, Jungpana, Kalej, Valley, Lingia, Makaibari Organic, Moondakotee, Nagri, Phoobsering, Risheehat, Singbulli, Snowview, Soom, Teesta, Valley, Tongsong and Tokdah [104]. The tea leaves for making Darjeeling tea may be picked in every season [105]. There are five tea flushes referring to the tea growing seasons (certain time periods) in Darjeeling region as follows:

#### Major tea flushes

- **The first flush tea** (spring tea, harvested in February–March).

It is the most sought after Darjeeling tea qualities. This tea has fresh aromas of flowers with full flavor of fine golden flowers and orange peels and is produced in an altitude of 3000 feet above sea level. It has a wonderfully complex flavor profile, such as a floral scent and lively character, very light/clear color and bright liquor, and aroma and mild astringency. The first flush tea is very expensive and produced in less quantity, hence, the demand outstrips the supply.

- The second flush tea (summer tea, harvested in May–June).

This tea is one of the most sought after teas from the Darjeeling region. It is so unique because it has the unique muscatel grape flavor due to the combination of the unique weather, topography, and the reaction to the infection of tea trees by small insects. This leads to the creation of the unique flavor and the trapins via the genes in the tea trees that only express themselves after attacked by insects that suck juices from the stems. The summer tea is usually heralded as the ‘champagne’ of teas, and drawn many connoisseurs. The second flush teas contain a higher amount of monoterpenoids including linalool, their oxides and geraniol compared with the rain flush tea [106].

- The third flush tea (autumnal tea, harvested in the autumn after the rainy season, i.e., October–November).

It has a somewhat less delicate flavor and less spicy tones, but the texture is full bodied with a darker or coppery color and a sparkling character.

#### Minor tea flushes

- Between flush tea

Tea leaves are harvested for 2 weeks in between the periods of the first and second flushes.

- Monsoon/rains tea (harvested in the rainy season between the second and third flushes)

It is less withered, consequently more oxidized, rarely exported, and usually sold at lower prices as the materials for preparing masala chai, a flavored tea beverage made by black tea with a mixture of aromatic Indian spices (ginger, cardamom, allspice, fennel seeds, star anise, etc.) and herbs (cinnamon and clove). Masala chai is the most popularly consumed beverage by Indian people.

#### Blend tea

- Darjeeling blend tea

It is a blend of Darjeeling teas which are created in every season and every tea garden in the Darjeeling region of India. Darjeeling blend tea has a unique flavor, full-bodied fragrance, bright tea liquor, and high quality [107].

- Darjeeling & Assama blend tea

A unique blend of fine orthodox tea leaves from Darjeeling and Assama, the world’s two best known tea planting regions. Darjeeling tea is renowned for its exquisite flavor and aroma; however, Assama tea is hugely popular for its strength and bright liquor. When combined both teas in an appropriate ratio, the perfect flavor and strength emerged. The characteristics of this blend tea are clear liquor, lovely amber color, light and delicate flavor coming from the Darjeeling tea coupling with the slightly malty and sweetish undertones of Assama tea at the same time [108].

### Assam black tea

Assam tea in India is shown in Fig. 13B. The Assam state of India, the world's largest tea-growing region and the second commercial tea production region after southern China, locates on both sides of the Brahmaputra River with an area of 78,440 km^2^ with clay soil rich in the nutrients of the floodplain. It belongs to the tropical monsoon rainforest climate, with generous rainfall (during the monsoon period, as much as 250 to 300 mm of rain per day, more than the most parts of India), high humidity (ranging from 56 to 90% in different seasons), and the maximum temperature at 35–38 °C in summer/rainy season and the minimum temperature at 6–8 °C in winter. As a result of the abundant rainfall and climate, huge Sal tree forests are found in the Assam state. Sal trees create greenhouse-like conditions of extreme humidity and heat, which provide an umbrella for avoiding sun shining directly onto tea bushes and contribute to the unique malty taste of Assam tea [109].

Assam tea possessing a beautiful ruby-amber hue, the biggest contribution to the world from Assam of India, named after the region of its production, is manufactured specifically from the plant *Camellia sinensis* var. *assamica* (Masters), which is considered as a variety of China’s version of *Camellia sinensis* var. *Sinensis* [110, 111]. Most of Assam tea bushes are grown at or near sea level in the valley of the Brahmaputra River, unlike Darjeelings and Nilgiris grown in the hillside/highlands of the Himalaya mountain and Western Ghats mountains, respectively. Assam tea is usually harvested twice a year. The first and second flushes are picked during March-June and October–November, respectively [112].

Assam tea, with a distinctive malty flavor and a bold and invigorating character, is a particular favorite for use as breakfast teas. The second flush Assam tea, the more prized “tippy tea” known for its creamy and flavorful liquor, is generally considered superior to the first flush tea. This high quality second flush black tea from the Assam region of Northern India is characterized by wonderfully full bodied and rounded in flavor of deep red liquor, which is robust and malty with a lingering jammy sweetness. However, most summer black teas from Assam are astringent and have a thick, malty and woody flavor profile. In addition, Assam region also produces smaller quantities of green and white teas with distinctive characteristics. Each year, the tea estates of Assam collectively yield approximately 680,000 tons, more than half of India's tea. Figure 13C shows the grades of Indian black tea [113].

### Ceylon (Uva) black tea

**Fig. 14:**
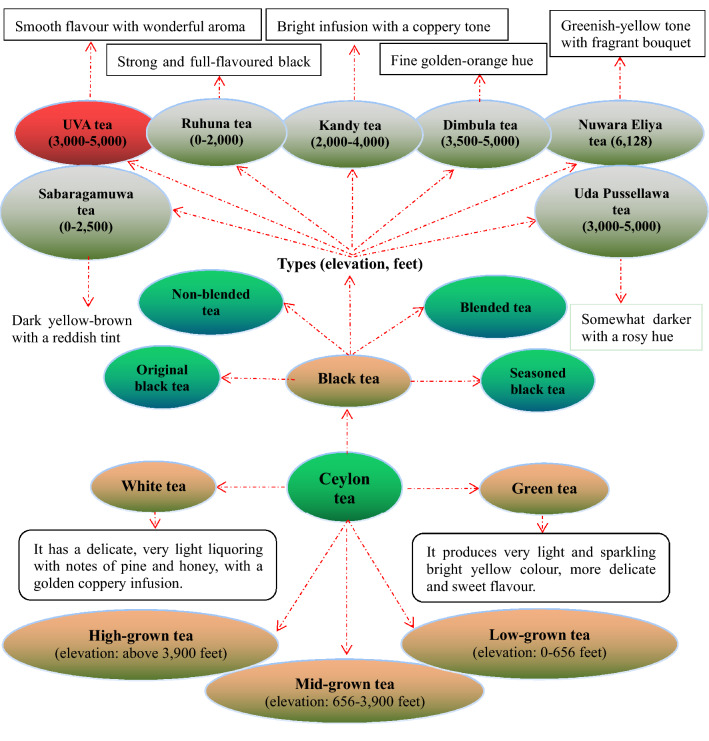
Sri Lanka black tea and Sri Lanka teas

Sri Lanka is the fourth-largest producer of tea in the world (Fig. 14). In 1824, a tea plant was brought to Ceylon by the British from China and planted in the Royal Botanical Gardens for non-commercial purposes. In 1867, the tea industry was born in Ceylon by the establishment of a tea plantation. In 1872, a fully equipped tea factory began operating in Kandy. In 1873, the first shipment of Ceylon tea (10 kg) arrived in London [114]. At present, tea manufacturing industry in Sri Lanka is one of the main sources of foreign exchange and accounts for 2% of Gross Domestic Product (GDP), contributing over $1.5 billion in 2013 to the economy of Sri Lanka, and employs over one million people. In 2014, its tea exported 318,000 tons, exceeding China’s tea trade volume (301,000 tons) [115]. This country can produce tea throughout the year. The tea tree-cultivating regions are mainly distributed in the central highlands, southern inland areas in the central highlands and southern inland areas, which provide a climate of humidity, cool temperatures and rainfall favoring the production of high-quality tea. At present, there are seven principal regions that grow pure Ceylon tea, including the Uva, Nuwara Eliya, Dimbula, Kandy, Uda Pussellawa, Ruhuna, and Sabaragamuwa geographic areas, each region producing a unique and highly valued tea [116, 117]. In addition, Ceylon tea is divided into three groups based on elevation, i.e., high-grown tea, mid-grown tea, and low-grown tea [118]. Most of Uva teas are manufactured using the orthodox process of production.

**Fig. 15:**
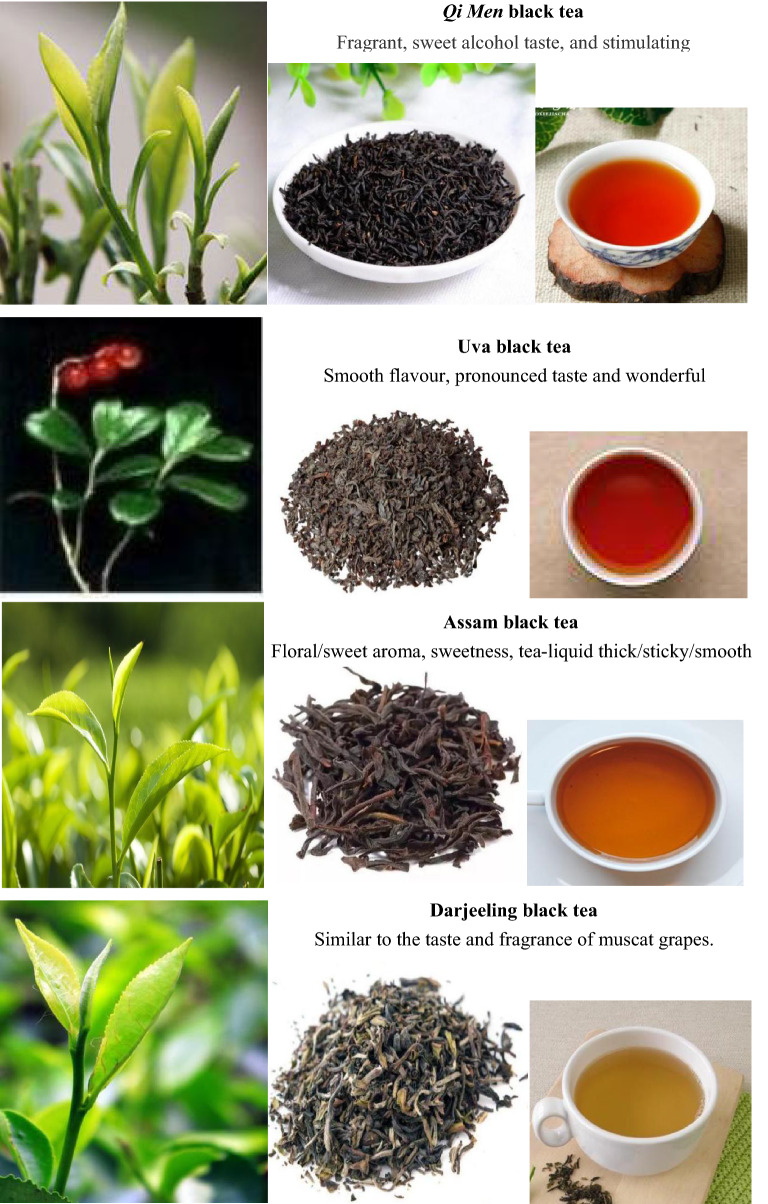
Four most famous black tea in the world. Data from https://image.so.com/i?src=360pic_strong&z=1&i=0&cmg=15484592.1427114627743071700.1627690750402.8748&q=%E7%BA%A2%E8%8C%B6%E5%9B%BE%E7%89%87%E5%A4%A7%E5%85%A8%E5%A4%A7%E5%9B%BE

The Uva area, on the eastern slopes of the central mountains of Sri Lanka, produces teas with a distinctive mellow flavor whose reputation stretches worldwide. The plants for making Uva tea grow at 3000 to 5000 feet above sea level in the Uva province. The tea leaves of the best Uva tea are plucked between June and September each year. The dry wind that blows towards Uva during this period gives Uva tea a copper colored infusion with a very smooth, pronounced taste and wonderful aroma. Uva tea is widely used in many blends. Uva is the most famous name among the seven main Ceylon tea-producing areas. Uva black tea, along with Chinese *Qi Men* tea and Indian Assam/Darjeeling tea, are considered as the four most famous black teas in the world (Fig. 15) [119]. Comparatively, the ten famous green teas in the world all originate from China (Fig. 10B).

### Black tea grade

Black tea, the most popular tea in the Western world, goes through full fermentation, in which the leaf color darkens to give it their black color. The flavor of black tea differs, ranging from flowery and fruity to spicy and nutty. The orthodox classification of black tea in the tea-producing countries has been fairly established based on the quality and processing methods. Indian black teas are divided into five grades according to the size of leaves, including whole leaves, broken leaves, fannings and dust. The Dust and FOP (flowery orange pekoe) is the lowest and highest grade, respectively. FOP is further divided into GFOP, TGFOP, FTGFOP, SFTGFOP and SFTGFOP1 from the low to high grades (Fig. 13C). Unlike Indian black tea, the grading criteria of *Qi Men* tea is more complicated. According to the appearance (such as the shape and color of tea leaves, integrity and consistency) and essentials (such as aroma, taste, tea liquor color and quality of the brewed tea leaves), *Qi Men* black tea is divided into ten grades: Hao ya A, Hao ya B, special grade, and grades 1–7. The highest grade is Hao ya A while the grade 7 is the lowest (Fig. 10E).

International black tea grades are as follows: CTC, crush tear curl; OP, orange pekoe; BOP, broken orange pekoe; FOP**,** flowery orange pekoe; TGFOP, tippy golden flowery orange pekoe; FTGFOP, fine tippy golden flowery orange pekoe; and SFTGFOP, super fine tippy golden flowery orange pekoe, such as Lapsang Golden Junmee which is the highest grade black tea in the world [120, 121]. In addition, the tea grade level is also assessed using many different analytical tools, for example, ultra-performance liquid chromatography-tandem mass spectrometry (UPLC-MS/MS), high-performance chromatography (HPLC), gas chromatography (GC), capillary electrophoresis (CE) and plasma atomic emission spectrometry, and GC × GC-FID, etc., based on the ingredients presented in tea [122–126].

## Components in tea

It is well known today that the two essential elements for human beings to survive on the earth are natural substances providing nutrients needed for our bodies and spiritual value attached these natural matters. The beneficial effects of tea on people are mainly based on the chemical ingredients in tea, as well as the psychic and cultural elements (such as those associated with tea ceremonies) presented in tea [127].

### Chemical components

**Fig. 16:**
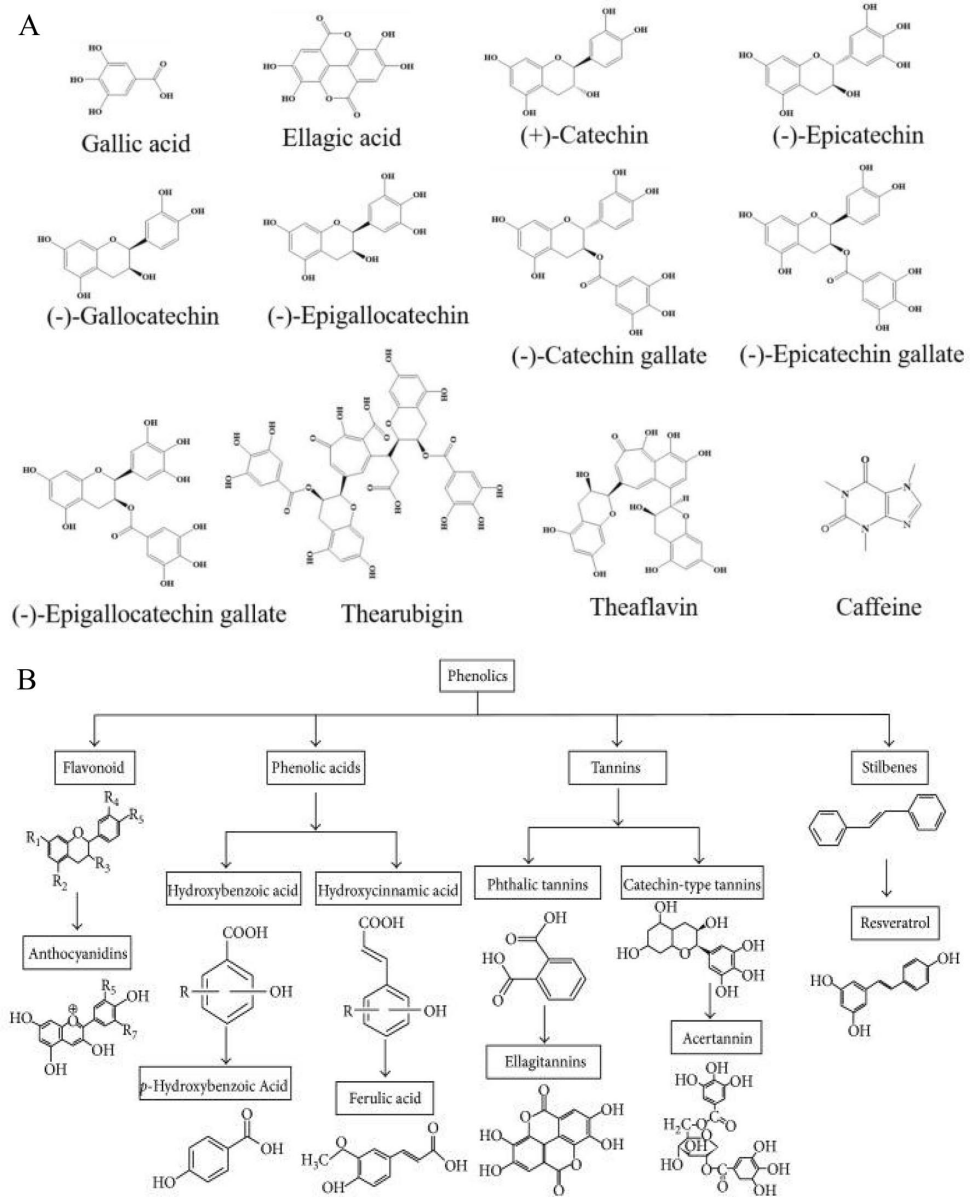
The chemical structures of several bioactive compounds in tea (**A**) and the classification and chemical structures of polyphenols in plant (**B**)

There are more than 700 different kinds of components in tea, including tea polyphenols, amino acids, organic acids, saccharides, lipoid substances, alkaloids, aromatic substances, vitamins, pigments, and enzymes, etc. The reputed health benefits of tea depend on what are in the tea. Chemical compositions of tea include inorganic substances (3.5–7.0%) and organic matters (93–96.5%). There are 27 kinds of mineral elements in tea such as P, K, S, Mg, Mn, F, Al, Ca, Na, Fe, Cu, Zn, Se, Fe, I, Mo, and Ge, etc. Organic compounds in tea include proteins (20–30%, but only 3.5% soluble in tea liquor), lipids (4–5%), 20 kinds of free amino acids (1.5–4%), carbohydrates (25–30%, but only 3–4% soluble in tea liquor), alkaloids, caffeine (2–4%), polysaccharides (0.1–0.5%), tea polyphenols (10–25%), organic acids, pigments (2–10% in black tea), chlorophyll (0.6–1.2%), carotene (7–20 mg%), cellulose (10–20%), aroma compounds, vitamins (B, C, E and U at 8–13, 50–300, 20–80 and 20–25 mg/100 g tea, respectively, and K at 300–500 U/g as well), saponin, and sterols, etc. [128]. Figure 16 A shows the major bioactive compounds in tea. Table 2 shows the elements in tea and their main functions on our body. Both aluminum and lead are regarded as causes of adverse effects on the body. According to the Chinese state criteria for food safety (GB2760-2011), the residual amount of aluminum should be less than or equal to 100 mg/kg food. According to the Chinese drinking water quality criterion (GB5749-2006), the lead concentration limit is less than or equal to 0.01 mg/L. Normally, the amounts of these two harmful elements in tea do not pose a threat on human health (Table 2).

**Table 2:** Constituents in tea and their functions on the body

Contents	Cantains (mg/10 g tea)	Human requirement (mg/adult/day)	Potential health effects
Potassium	140–300	1000–3400	Maintain body fluid balance
Magnesium	1.5–5	310–420	Maintain normal glucose metabolism
Manganese	3.8–8	2–5	Maintain the activity of enzymes related to reproduction and bone
Fluorine	1.5–5	1.5–4	Prevent dental decay
Calcium	3–4	800	Contribute to bone growth
Sodium	2–8	2000	Maintain body fluid balance
Sulfur	5–8		Contribute to circulating metabolism
Iron	0.6–1	15–20	Related to hematopoiese
Copper	0.5–0.6	2	Participate in the action of enzymes
Silicon	0.2–0.5	20–50	Improve bone development
Zine	0.2–0.4	15	Improve growth and development
Selenium	Trace	0.05–0.25	Enhance immune function
Nickel	0.05–0.28	0.3	Maintain normal metabolism
Aluminum	0.4–1	< 40	Not necessary
Lead	Minimal amount	< 0.03	Not necessary
Polyphenols	3461–3644 (green tea)		Antioxidant, antibacteria, antivira, anticancer, lowering blood lipid [131]
	1290–2176 (black tea)		
Tea pigment	200–1000 (black tea)		Antioxidant, lowering blood lipid [132]
Flavonoid	276–375		Antioxidant, anticancer [133]
Tannins	354–591 [129]		Combating oxidative stress, antipathogenic microorganism [134]
Caffeine	200–400		Stimulating the brain and the heart, diuresis
Polysaccharid	10–50		Regulating immune function, lowering blood glucose [135]
Cellulose [130]	1000–2000	25,000	Helps digestion, reduces blood lipids, and inhibits lipid peroxidation [136]
Chlorophyll	60–120	Very little	Antioxidant, anticancer [137]
Carotene	0.7–2 (green tea)	1000 U	Preventing blindness and cataract, anticancer
Vitamin B	0.8–1.3	1.5–2	Benefit for the skin and nerves
Vitamin C	5–30	50–100	Anti-scurvy, increase immune function
Vitamin E	2–8	10	Anti-oxidant/aging, balance lipid metabolism

Up to know, 164 compounds have been identified from dark tea. The main components are catechins (epicatechin, epigallocatechin, epicatechin gallate, and EGCG), flavones, phenolic acids, phenylpropanoids, terpenoids, caffeine, pyrimidines, alkanes, aryl compounds, fatty acids, anthraquinones, and esters, etc. [138]. The major chemical compositions of green tea plant include polyphenolic compounds, predominantly catechins, proteins, carbohydrates, vitamins (C, B, and E), xanthic bases (caffeine and theophylline), pigments, minerals, and trace elements [10, 139]. More than 100 chemical components have been isolated and identified from oolong tea, and (–)-Epigallocatechin-3-gallate present in this tea can prevent the growth of cancerous cells [140]. Theanine and polyphenols in tea are closely related to tea flavor and aroma, therefore, they are usually used as the standards for judging tea quality [141]. Polyphenol compounds in tea are considered the main functional components contributing to human health [142].

### Polyphenolic compounds

Although thousands of various biological compounds are found in tea, research results have demonstrated that the health benefits of tea are related to tea polyphenols, in addition to the secondary metabolites such as flavan-3-ols, phenolic acids, purine alkaloids, condensed tannins, hydrolysable tannins, saponins, flavonols, and their glycoside forms, etc. [143]. In fact, polyphenol substances (Fig. 16B) exist widely throughout the plant kingdom, and therefore, they are an integral part of the human diet, as dietary polyphenols [144]. Tea tree belongs to plant family, but tea is processed plant leaves by withering, rolling, fermentation, post-fermentation and roasting. Each type of tea has its own critical manufacturing process and possesses unique chemical compositions, flavor and bioactivities.

**Fig. 17:**
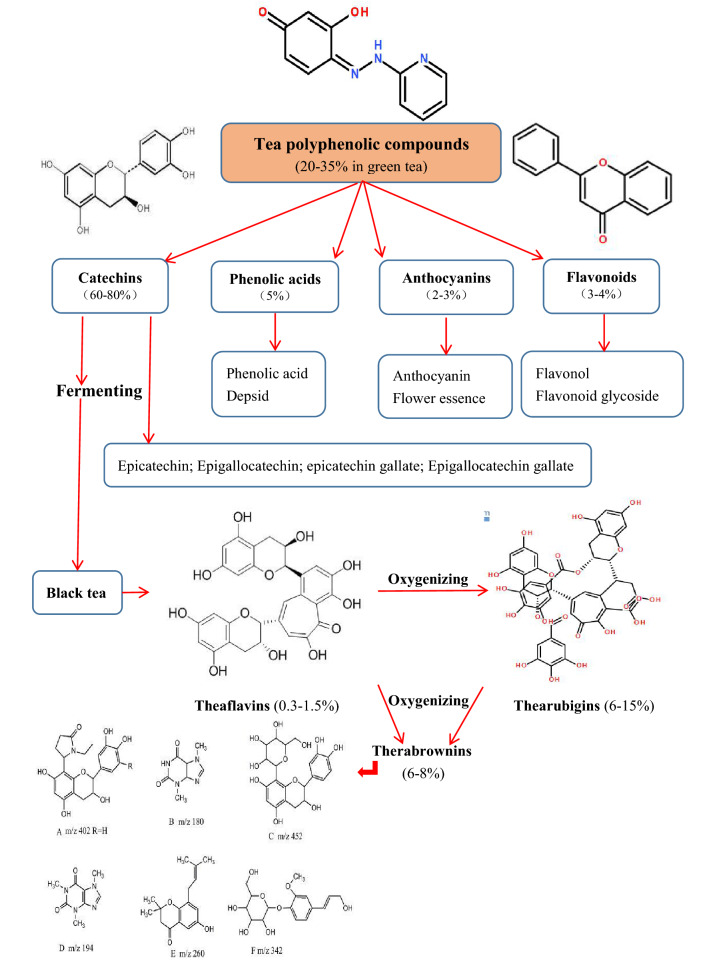
Polyphenol-related compounds in tea

According to processing methods, mainly in the degree of fermentation, teas are generally divided into green tea, black tea, oolong tea, and scented tea which is green tea, black tea, or oolong tea processed with fresh flowers. Hence, polyphenol substances in tea leaves undergo some changes. For example, catechins (epicatechin, epigallocatechin, epicatechin gallate, and EGCG), the main components of tea polyphenols accounting for about 70% of polyphenols in tea leaves, are converted to theaflavin, thearubigins, and therabrownins, the oxidized derivatives of catechins during fermentation (Fig. 17) [145]. Oxidized derivatives of catechins are considered to be the major bioactive components of black tea, especially responsible for the antioxidant actions [146].

The absorption spectrum of visible light is different among the three classes of substances (theaflavin, thearubigins and therabrownins) in black tea using the spectrophotometric method. The absorption peaks of theaflavins are at 380 nm and 460 nm, and theaflavins at 360–380 nm. However, the absorption peak of therabrownins was not found, indicating that therabrownins belong to the substances of different properties and structures [147, 148]. Recently, it was found that tea polyphenols, EGCG from green tea and theaflavin digallate from black tea, might be the lead compounds in prophylaxis and treatment of COVID-19 as suggested by molecular docking of tea polyphenols with some of the possible binding sites of the severe acute respiratory syndrome coronavirus 2 (SARS-CoV-2) proteins [149]. A catechin, EGCG, binds to the S1 ubiquitin-binding site of papain-like protease (PLPro) and might inhibit the protease function and abrogate SARS-CoV-2’s inhibitory function on the ubiquitin–proteasome system and interferon-stimulated gene expression [150].

Although catechins, theaflavins and thearubigins are considered to be the main active components of tea, increasing evidence indicated that the beneficial health effects of tea might result at least in part from the breakdown products of the polyphenols formed in the gut [151]. Because of the low bioavailability of polyphenols and the gut microbiota which are closely correlated to human health, most of polyphenol substances remain in the gut for some time and metabolize to a variety of derivatives by intestinal flora, thus altering the intestinal micro-ecology, before showing their biological activities [152]. For example, tea catechins are transformed into hydroxyphenyl-γ-valerolactones, and then further metabolized to smaller phenolic acids via the gut flora. At the same time, the effects of tea on some beneficial gut microbiota for improving human health have been studied along with the new technology development [153]. Theabrownin in the *Pu'er* tea altered the gut microbiota, reduced hepatic cholesterol, and decreased lipogenesis in mice and humans through suppressing microbes associated with the bile-salt hydrolase activity [154]. Polyphenols, polysaccharides, and teasaponin, the major active components in tea, can alleviate the gut microbiota dysbiosis induced by high-fat diet [155, 156]. Polyphenols enhance energy conversion by boosting the gut-microbiota-dependent mitochondrial tricarboxylic acid cycle and urea cycle in rats [157].

## Discussion

Nowadays, with the aging of populations worldwide, more people suffering from non-communicable, incurable diseases, and/or poor health, which poses a considerable challenge to clinicians and the societies. CAM provides an array of treatment modalities for health promotion. CAM therapy can be divided into two major strategies, namely, herb-based CAM therapy and non–herb based CAM therapy [31]. Tea may be the first plant medicine or CHM, a simple herb-based CAM therapy, which has been used for improving human health by Chinese ancestors since 9000 ago, as *Shen Nong* (神农) who discovered tea was a clan originated from the agricultural tribes in the ancient *Wu Ling* area about 9000 years ago in China [158]. Therefore, tea has been loaded with a lot of Chinese history, culture and medicine, and affected the world from the past to the present. It is not only a beverage, but also contains so many stories, culture and spirit, besides the chemical elements of tea. Because tea has the potential to improve our physical health [159, 160], it has attracted the attention of many researchers and the food industries besides tea-drinkers. They attempt to find single compounds as pharmaceutical candidates or develop new high-functional foods from tea by means of separation and purification technologies, which may separate the integral effects of the multi-bioactive ingredients in tea, as well as the cultural and immaterial values attached to the tea. Science, culture and spirit/faith are the foundation stones of human evolution and civilization development. Even in the current levels of human civilization, they are still the foundation for building a harmonious society. None of them is dispensable. Therefore, in the era of aging population and super-society of the world, the importance of tea and tea-drinking become more and more highlighted.

The common aspiration of people around the world is health, health, and health, as well as longevity, longevity, and longevity! Admirable vibrant health involves the well-being of the body, mind and spirit, and the connections among them. A person’s status of health, therefore, is synthetically determined by four areas of health: (1) physical health, meaning that one can move through the world the way he likes, without disease, ailment or discomfort; namely, the ability to exist and the body to operate; (2) spiritual health, which is not necessarily about religion or faith, but it is related to one’s ability to know and pursue purposes, without feeling detached, lost or confused; (3) mental health, defined as one’s abilities to think clearly, focus, and perform cognitive tasks, without feeling stressed, scattered or overwhelmed; (4) emotional health, which is one’s abilities to give and receive love, make connections and have belonging, as well as mastering one’s feelings and behaving in such a way that invites others into one’s inner circle [161–163].

Human health can be obtained from daily diet (food and drink), life style, and culture, which may be partially supplied by tea. The beneficial effects of tea on human health are obtained through three ways. (1) Constituent-associated way, such as the anxiolytic effects of theanine [164, 165] and antiviral activity of tea polyphenols [166]; (2) Culture-associated way, drinking tea has rich cultural connotation, as people have cultural needs and dependence; (3) Constituent/culture-associated way, as man is a heteroion of material and spirit, both of which have impacts on human health [167]. Drinking tea may produce positive influences on both the material and spirit parts. For example, tea-drinking processes can make people enjoy the cheerful feelings, and at the same time, theanine, a nonproteinic amino acid special to tea, has positive effects on the emotional status [168].

The pursuit of some Chinese people, especially ancient intellectuals, on spirit is always of greater importance than that of matters. They might put some simple things in very high spiritual/cultural values and treated their daily or physiological needs as a very sophisticated pastime or affair. In China, ancient intellectuals especially emphasized on the specifications and elegance of their daily performance, for examples, from diet to food culture, from alcohol to wine culture, from medicine to medical culture, from tea-drinking to tea culture, and even from sex to sexual culture. Because the social or personal wealth was in shortage to meet the needs of life and the methods and approaches for understanding nature and matters were limited, many people in the old China, especially the literati class with hidden, elegant and self-disciplined sentiments had to seek spiritual satisfaction instead. Man can reach the highest level of spiritual pursuit, although he may never touch the boundary in the pursuit of wealth. At this very moment, the COVID-19 morbidity and mortality rates in the USA are among the highest in the world, despite the fact that it is one of the richest countries with the best science and technology in the world. It is very surprising, up to the present moment, that there are so many Americans who refuse to wear masks and are still fond of parties as COVID-19 pandemic spreads rapidly far and wide in USA. There are many reasons why this is happening. To begin with, unlike Chinese culture, American culture overly emphasizes on individual rights and freedom. In fact, mask-wearing and respecting the necessary social distancing can effectively stop the human-to-human transmission chain of this virus via the respiratory tract [169–173].

When tea ceremony is understood and practiced to foster harmony in humanity, promote harmony with nature, discipline the mind, quiet the heart, and attain the purity of enlightenment, the art of tea becomes “*Cha Dao*” (tea ceremony)—achieving the spiritual realm of the unity of man and nature and striving for progress with self-cultivation. Therefore, tea ceremony is mostly a simplistic mode of aesthetics, but also provides subtle insights into ethics, metaphysics, and a sense of focus and concentration under the influence of the tasty tea. At present, human beings face serious environmental and social problems, especially during the COVID-19 pandemic, and the wisdom of the Chinese ancient people will prove useful. Moreover, polyphenol substances can provide health benefits. Epigallocatechin-3-gallate from green tea and theaflavins from black tea have exhibited antiviral activities against various viruses, especially positive-sense single-stranded RNA viruses, and immunomodulatory competence [174]. Caffeine and its biosynthetic intermediates, theophylline and theobromine, in tea infusions have anti-inflammatory, anti-microbial, anti-oxidant and anti-tumor activities [16]. Copper, iron, manganese, selenium and zinc in tea are the integral part of the innate immune response [175]. It has been demonstrated that vitamins, minerals and other bioactive compounds in tea have potential roles in interfering with spike glycoproteins and angiotensin converting enzyme 2, thus mitigating the spread of the unprecedented COVID-19 pandemic [176]. Molecular dynamics simulations and Molecular Mechanics-Poisson Boltzman Surface Area (MM-PBSA) studies suggested oolonghomobisflavan-A as a potential bioactive molecule to act as an inhibitor of the Mpro of SARS-CoV-2 [177].

Tea-drinking, in fact, replenishes the body with adequate water, which is beneficial for improving health. Moreover, when adding the cultural elements to tea-drinking behaviors, the overall well-being of the tea drinkers will be greatly improved. This transcendent thought patterns of the Chinese people stems from our ancestors' worship and reverence of nature and life. Man is also a part of nature. Among the cultures created by China’s ancestors, tea culture has been fully accepted by people around the world. At the same time, TCM with rich cultural connotations is also gradually going to the world. The improvement of human health requires both modern medical science and traditional medicine, therapies and ideas. The beneficial effects of tea and tea therapy for health care have been supported not only by the TCM theory, but also by modern sciences which enable testing of tea constituents in animal and cell experiments and clinical studies. Recently, it has been demonstrated that green tea, oolong tea, black tea, white tea and dark tea can activate the Grand Solar meridians (*Tai Yang Jing*, 太阳经), Middle Solar meridians (*Yang Ming Jing*, 阳明经), Little Solar meridians (*Shao Yang Jing*, 少阳经), Grand Lunar meridians (*Tai Yin Jing*, 太阴经), and Middle Lunar meridians (*Jue Yin Jing*, 厥阴经), respectively, as visualized by infrared photography [178]. The human meridian (*Jing Luo*, 经络) system, which has twelve formal meridian channels going through the respective organs, carrying fluid and energy, and generating thermal effects, is a key foundation of the TCM theory.

## Summary

The main points about tea and tea drinking are summarized as follows:

Overview

1. China is the homeland of tea trees and tea drinking.
2. Black tea and green tea are the most popular worldwide.
3. Uva tea is the most famous black tea in the world.
4. *Xi Hu Long Jing* tea is the most famous green tea in the world.
5. Tea is consumed more than any other man-made drinks in the world.
6. The finest tea is grown at altitudes of 900 to 2100 m above sea level.
7. China has a very rich and brilliant tea culture, which goes far beyond tea itself.
8. Theanine and polyphenols are usually used as the standards for judging tea quality.
9. Tea is best stored in a refrigerator at about 5ºC, if you want to maintain its flavor for a long time.
10. Black tea and dark tea are the most oxidized (fermented), green tea and white tea are not oxidized, while oolong tea is partially oxidized.

How to brew tea

1. Good quality spring water or rainwater from autumn is the best one for brewing tea.
2. The water temperature for brewing black tea should be around 90ºC, and for about 3 min of infusion.
3. The water temperature for brewing green tea should be around 80ºC, for about 2 min of infusion
4. If the water is too hot, nutrients in tea will be destroyed and the tea liquor may taste badly.
5. If the water temperature is too low, tea leaves will not fully open up, tea flavors and bioactive ingredients will not be fully extracted, and you will not see the beautiful scene of infused tea leaves.
6. The purple clay wares made in *Yi Xing* and *Jing De Zhen* in China are among the best choices for brewing tea.

Bioactive ingredients in tea and their beneficial effects on health

1. Theaflavins and thearubigins are the main active components in black tea [179].
2. Polyphenols such as catechins are the main active components in green tea [180].
3. The art of serving and drinking tea plays a cultural/spiritual role in the drinkers.
4. Tea can improve both human physical and mental health, and even defer senility.
5. Tea consumption reduces the risks of mortality, cardiac death, coronary artery disease, stroke, and type 2 diabetes mellitus [181].
6. Other health functions include antioxidant, anti-inflammatory, immuno-regulatory, anti-cancer, anti-obesity, anxiety reduction, cognitive enhancement, anti-bacterial, anti-viral, and anti-COVID-19 activities, etc.
7. It has been found that catechins from oolong tea can improve the ovarian functions in polycystic ovary syndrome mice [182]

Some matters needing attentions when drinking tea

1. Tea may affect the therapeutic effects and absorption of some medicine [183, 184].
2. Drinking overly strong tea can inhibit the absorption of iron in the gut [185].
3. Ready-to-drink liquid teas in the market may not be healthy beverages.
4. The observed safe level of 704 mg EGCG/day might be considered for tea preparations in beverage form based on human adverse event data [186].
5. Tea may cause hepatic and gastrointestinal disorders, especially when consumed on an empty stomach [187].
6. It is not proper for pregnant women, especially in the last trimester of pregnancy, or breast-feeding women to drink tea [188].
7. Generally, spring, summer, autumn, and winter are more proper for drinking scented tea, green tea, oolong tea, and black tea, respectively. The same goes for the time of the day from the morning to evening.
8. Tea of the Benifuki variety (Japan tea) picked in September–October is preferable for consumption by children and pregnant women [187, 189].
9. Hot tea increases the risks of esophageal and gastric cancers when the temperature of intake is more than 55–60 °C [190, 191].
10. United States Pharmacopeia included a cautionary labeling requirement for Powdered Decaffeinated Green Tea Extract products that reads as follows: Do not take on an empty stomach. Take with food. Do not use if you have a liver problem and discontinue use and consult a healthcare practitioner if you develop symptoms of liver trouble, such as abdominal pain, dark urine, or jaundice (yellowing of the skin or eyes) [192].

To sum up, proper tea consumption has greater benefits than harm to human health [160, 193].

## Conclusions

Tea, a simple herb-based CAM therapy, has been widely used as an auxiliary means to maintain people’s physical and mental health, and has brought social benefits, especially for the elderly and urban residents. Because the oldest tea trees in the world, tea leaf fossil from 35 million years ago, and abundant historical literature of tea are found in China, undoubtedly, China is the birthplace of tea. Tea consumption began in ancient China over 5000 years ago and spread into every aspect of the society through the tea culture, influencing the course of the Chinese and world histories. Today, tea has become a very important part of the way of life of about 3 billion people worldwide. People have two attributes and two needs, namely, social and biological attributes, and material and cultural/spiritual needs. Unlike TCM, modern medicine usually emphasizes on human biological attributes and material needs, but often neglects human social attributes and cultural/spiritual needs. It is well known that health maintenance needs both materials and spiritual elements. Tea drinking not only benefits human physical health via bioactive compounds present in tea, such as polyphenols in green tea and theaflavin and thearubigins in black tea, but also improves human mental health via tea culture and tea arts. The later can further improve human physical health status. Mental joy and spiritual satisfaction make people happy. Besides, some compounds of tea such as theanine can directly excite human emotion.

Tea is indeed relatively inexpensive and good for the human health.

## Data Availability

All the data used to support the fndings of this study are available from the corresponding author upon reasonable request.

## Abbreviations

BOP: Broken orange pekoe
CE: Capillary electrophoresis
CHM: Chinese herbal medicine
CIS: Commonwealth of Independent States
COVID-19: The coronavirus disease 2019
CTC: Crush tear curl
EGCG: Epigallocatechin gallate
FOP: Flowery orange pekoe
FTGFOP: Fine tippy golden flowery orange pekoe
GC: Gas chromatography
GDP: Gross domestic product
HPLC: High-performance chromatography
MM-PBSA: Molecular Mechanics-Poisson Boltzman Surface Area
NPQ: The national peculiar quintessence
OP: Orange pekoe
SCC: Science and Civilization in China
SFTGFOP: Super fine tippy golden flowery orange pekoe
TCM: Traditional Chinese medicine
TGFOP: Tippy golden flowery orange pekoe
UPLC-MS/MS: Ultra-performance liquid chromatography tandem mass spectrometry
